# Supplementary material for: A molecular atlas reveals the tri-sectional spinning mechanism of spider dragline silk
Source: Nat Commun. 2023 Feb 15;14:837. doi: 10.1038/s41467-023-36545-6 (PMC9932165; doi:10.1038/s41467-023-36545-6)
Supplement: Supplementary file 1 — Supplementary Information [file 41467_2023_36545_MOESM1_ESM.pdf]

A molecular atlas reveals the tri-sectional spinning mechanism of spider dragline silk

Wenbo Hu[1,#], Anqiang Jia[1,#], Sanyuan Ma[1], Guoqing Zhang[1], Zhaoyuan Wei[1], Fang Lu[1], Yongjiang Luo[1], Zhisheng Zhang[2], Jiahe Sun[1], Tianfang Yang[1], TingTing Xia[1], Qinhui Li[1], Ting Yao[1], Jiangyu Zheng[1], Zijie Jiang[1], Zehui Xu[1], Qingyou Xia[1,]*, Yi Wang[1,]*

1 State Key Laboratory of Silkworm Genome Biology, Biological Science Research Center, Southwest University, Chongqing 400715, China.

2 School of Life Sciences, Southwest University, Chongqing 400715, China.

[#]These authors contributed equally to this work.

*Corresponding authors.

E-mail: yiwang28@swu.edu.cn, xiaqy@swu.edu.cn

Supplementary Figures

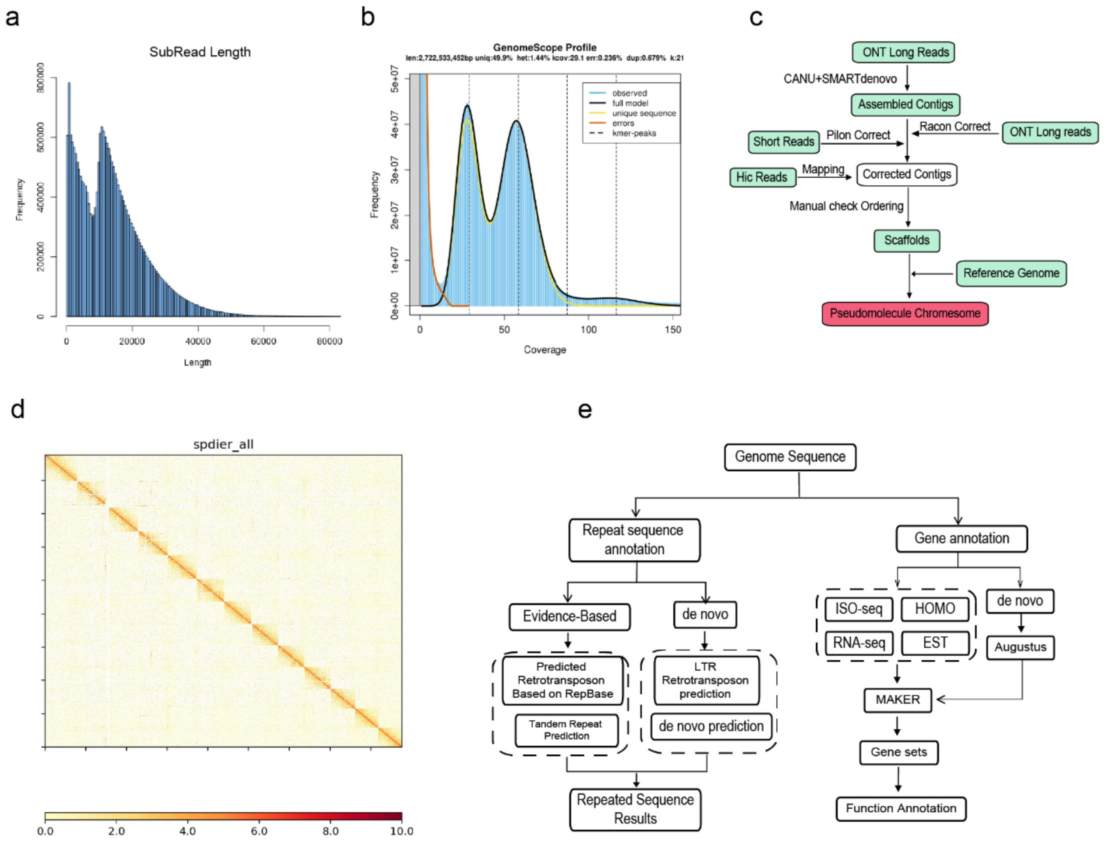

Supplementary Fig. 1: Brief information on the female *T. clavata* genome assembly and annotation. **a**, Distribution of the ONT reads length. **b**, The estimated genome size of *T. clavata* spider based on K-mer analysis. **c**, Brief pipeline of genome assembly based on the ONT, Illumine, and Hi-C reads. **d**, Hi-C interaction map of 13 pseudochromosomes of *T. clavata* genome. The interaction intensity increases from yellow to red. **e**, Brief pipeline of genome annotation based on homology data and de novo predictions.

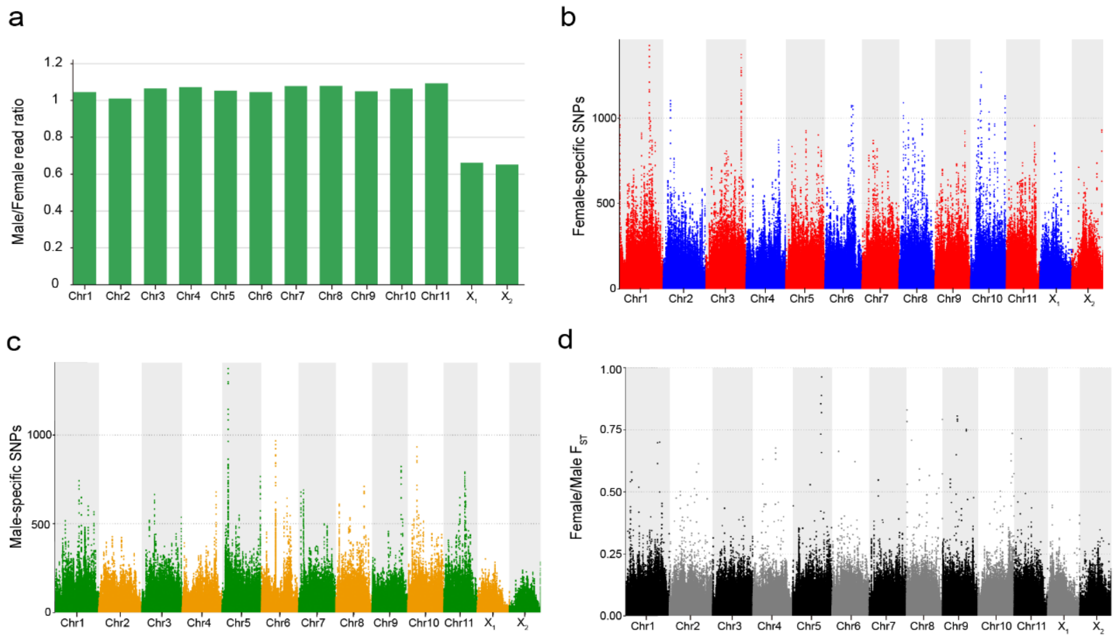

Supplementary Fig. 2: Sex chromosome identification and analysis in *T. clavata*. a, The read ratio of male/female based on Pool-seq for each chromosome. **b**–**d**, Manhattan plots for female-specific SNPs (**b**), male-specific SNPs (**c**), and female/male F_{ST} (**d**).

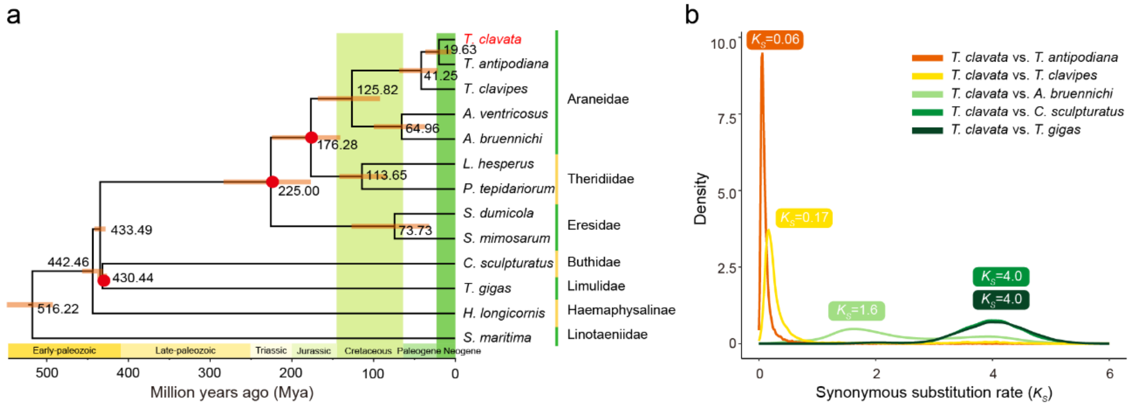

Supplementary Fig. 3: Phylogeny of the *T. clavata* spider. a, Phylogenetic tree of *T. clavata* and other 12 species (*T. antipodiana*, *T. clavipes*, *A. ventricosus*, *A. bruennichi*, *L. hesperus*, *P. tepidariorum*, *S. dumicola*, *S. mimosarum*, *C. sculpturatus*, *T. gigas*, *H. longicornis*, *and S. maritima*). The red circles represent the time correction points from the TimeTree (http://www.timetree.org/), and the numbers on the nodes represent the divergence time. **b**, Distribution of the synonymous substitution rates (K_s) of homologous genes between *T. clavata* and five arthropod species (*T. antipodiana*, *T. clavipes*, *A. bruennichi*, *C. sculpturatus,* and *T. gigas*). Five peaks (K_s = 0.06, 0.17, 1.6, 4.0, and 4.0) of the K_s distribution indicate the divergences of *T. clavata* and *T. antipodiana*, *T. clavipes*, *A. bruennichi*, *C. sculpturatus,* and *T. gigas*.

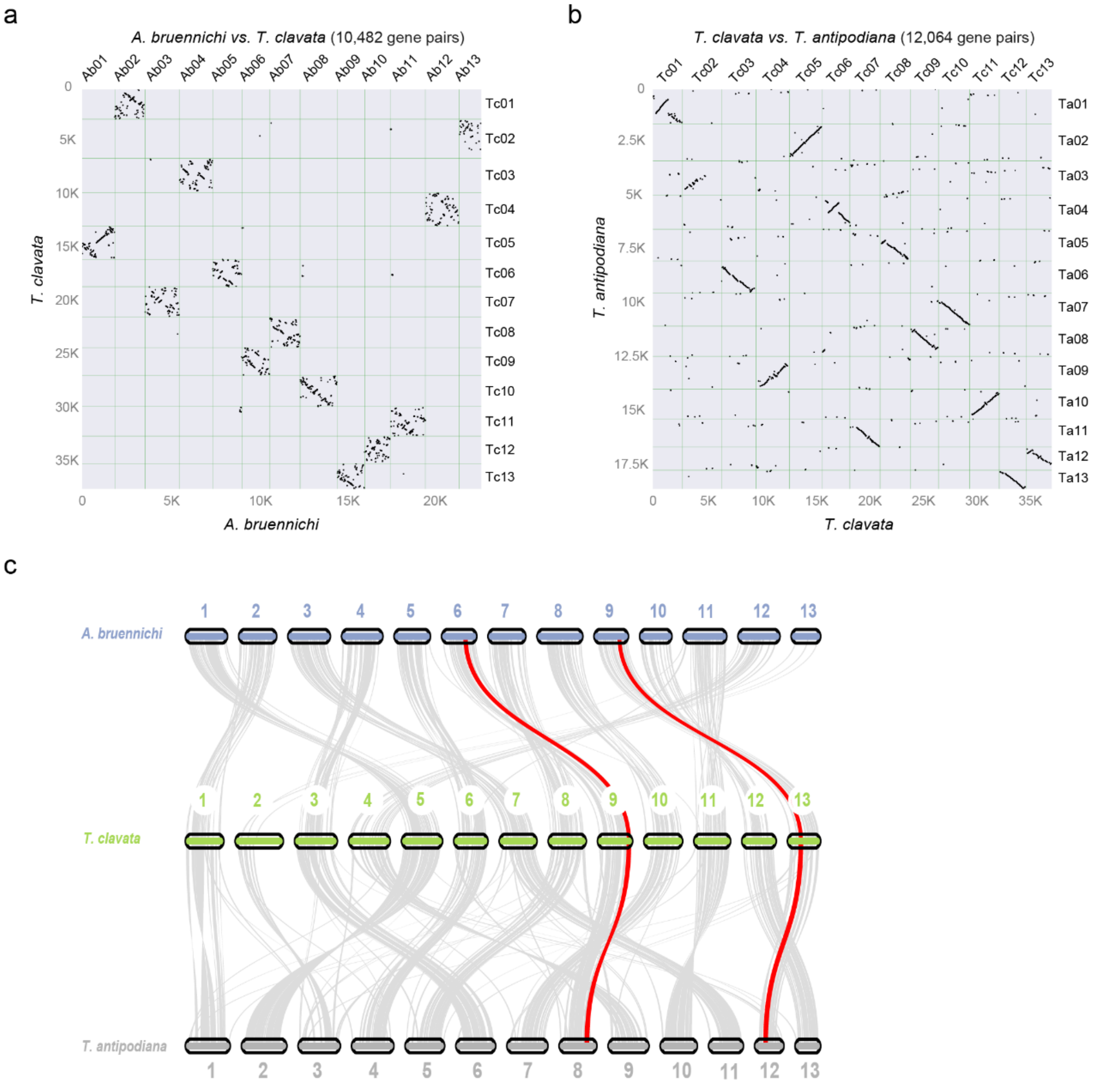

Supplementary Fig. 4: Collinearity relationships of genes. a, **b**, Dot plot of orthologous gene pairs for *A. bruennichi* and *T. clavata* (**a**), *T. antipodiana* and *T. clavata* (**b**). Each dot represents an orthologous gene pair. Consecutive dot arrangements indicate higher collinearity between species. **c**, Gene collinearity between *A. bruennichi*, *T. antipodiana,* and *T. clavata*. The gray lines depict homologous genome blocks between species. The red lines represent *Hox* clusters.

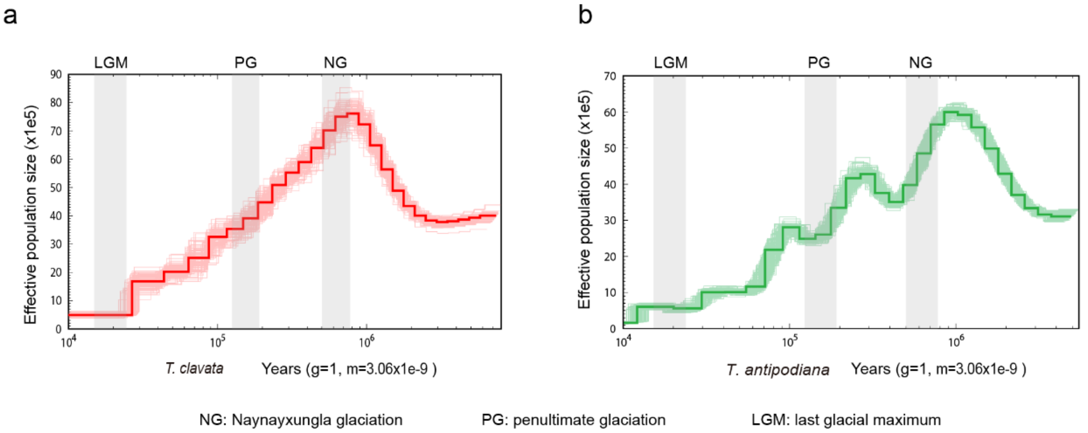

Supplementary Fig. 5: Demographic history. **a**, **b**, Population history of *T. clavata* (**a**) and *T. antipodiana* (**b**). The y axis corresponds to the effective population size. NG, Naynayxungla glaciation. PG, penultimate glaciation. LGM, last glacial maximum. Mutation rates were estimated using the formula $\mu = K_s/2T$, where $K_s = 0.06$ and T = 19.63 Mya were employed as the synonymous substitution rates and the divergence time. The synonymous mutation rate was estimated as $3.06 \times 10e^{-9}$ with a generation time of one year.

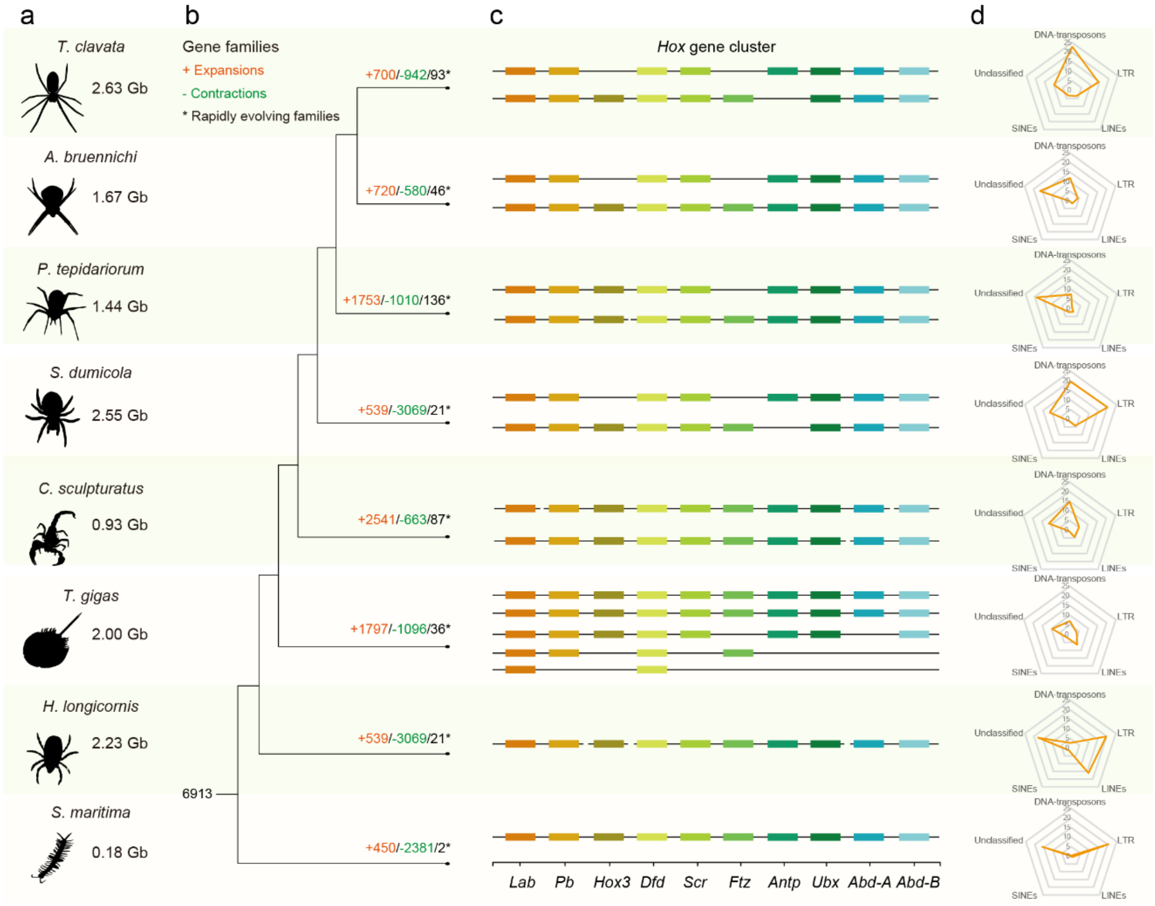

Supplementary Fig. 6: Gene family expansion and contraction, schematic representation of *Hox* clusters, and genomic structure in eight species. a, Mode charts and genome size statistics for eight species. **b**, Expansion, contraction, and rapidly evolving gene families. The number 6,913 represents the total number of orthologous gene families shared by eight species. **c**, *Hox* cluster distribution. Different *Hox* genes are labelled with colored boxes. **d**, Radar chart of TE content. The *T. clavata* and *S. dumicola* show great differences in the composition of repetitive sequences. The coordinates represent the ratio of each TE category to the total length of the corresponding genome assembly.

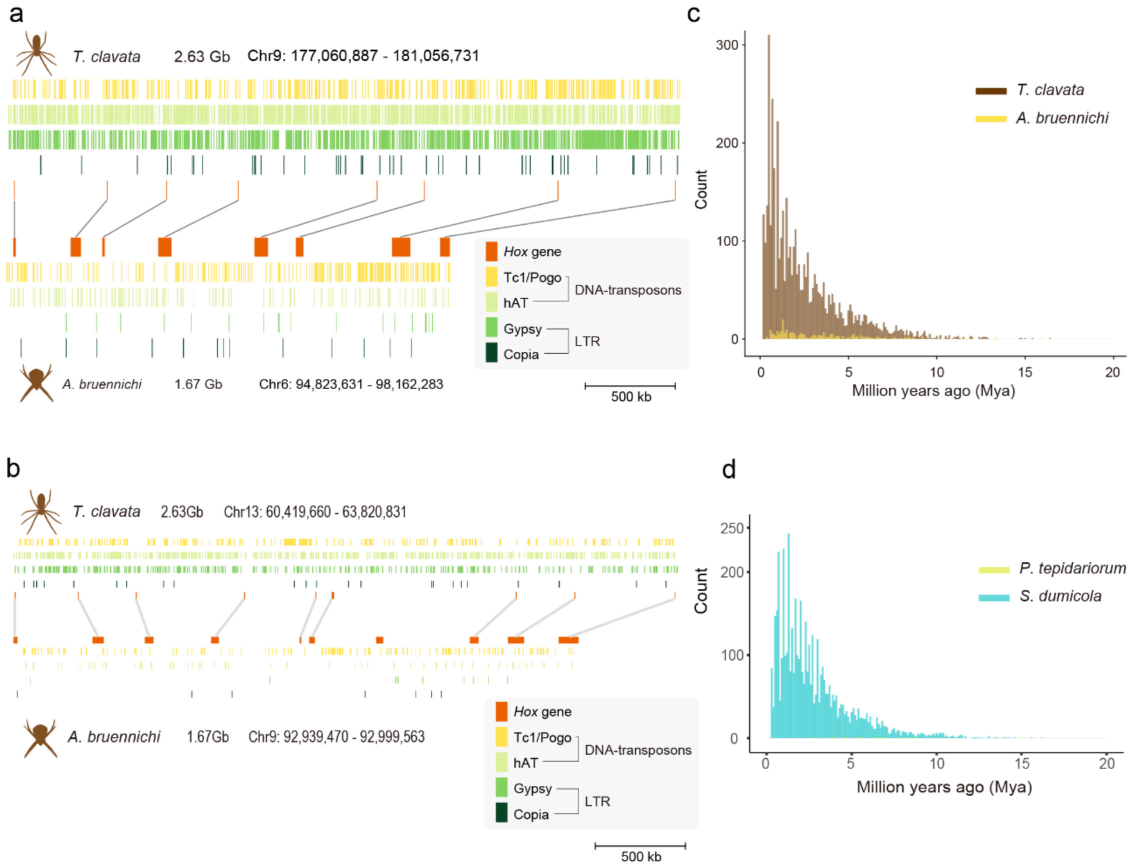

Supplementary Fig. 7: Transposon distribution. a, **b**, Insert distribution of the DNA (Tc1/Pogo and hAT) and RNA transposons (LTR: Gypsy and Copia) in two *Hox* clusters of *T. clavata* and *A. bruennichi*. Shortlines with different colors represent the different TE categories. **c**, **d**, Histogram of LTR insert time for *T. clavata* and *A. bruennichi* (**c**), *P. tepidariorum* and *S. dumicola* (**d**). The y axis represents the LTR retrotransposon counts.

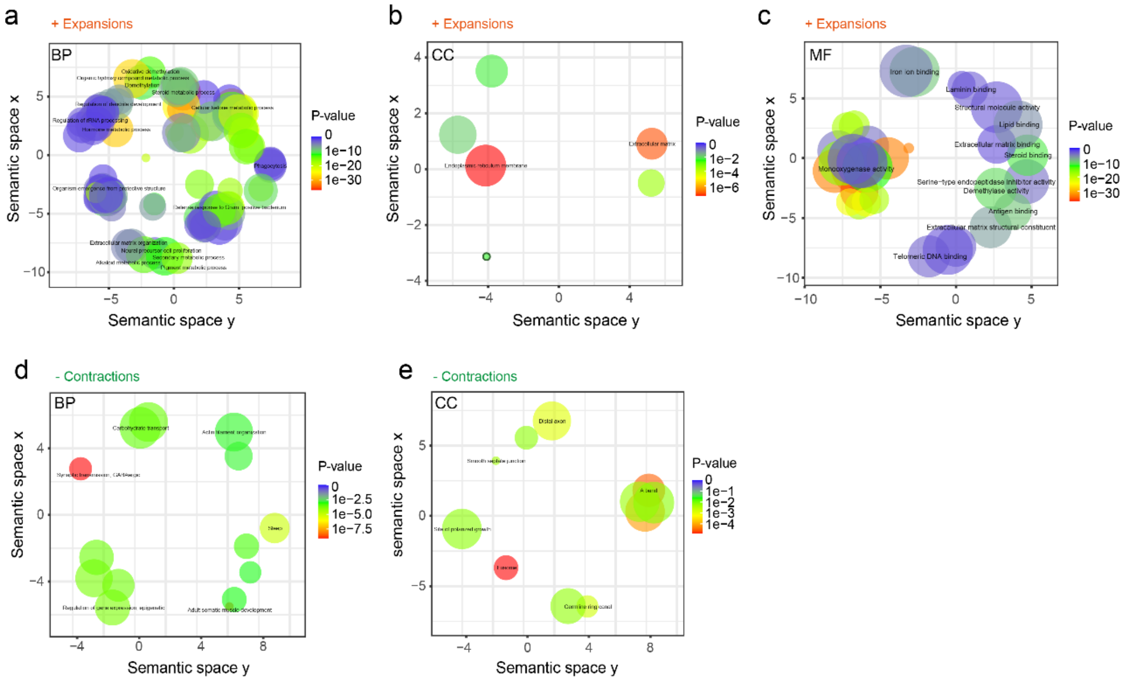

Supplementary Fig. 8: GO annotations of expanded and contracted gene families in *T. clavata*. **a**–**c**, Expanded gene families were enriched in the GO terms of the biological process (BP) (**a**), cellular component (CC) (**b**), and molecular function (MF) categories (**c**). **d**, **e** Contracted gene families were enriched in the GO terms of the BP (**d**) and CC (**e**) categories but not the MF category. *P*-value assigned to each enriched GO term was calculated by using Fisher's exact test. *P*-value < 0.05 was set as the criteria for screening significantly enriched GO term.

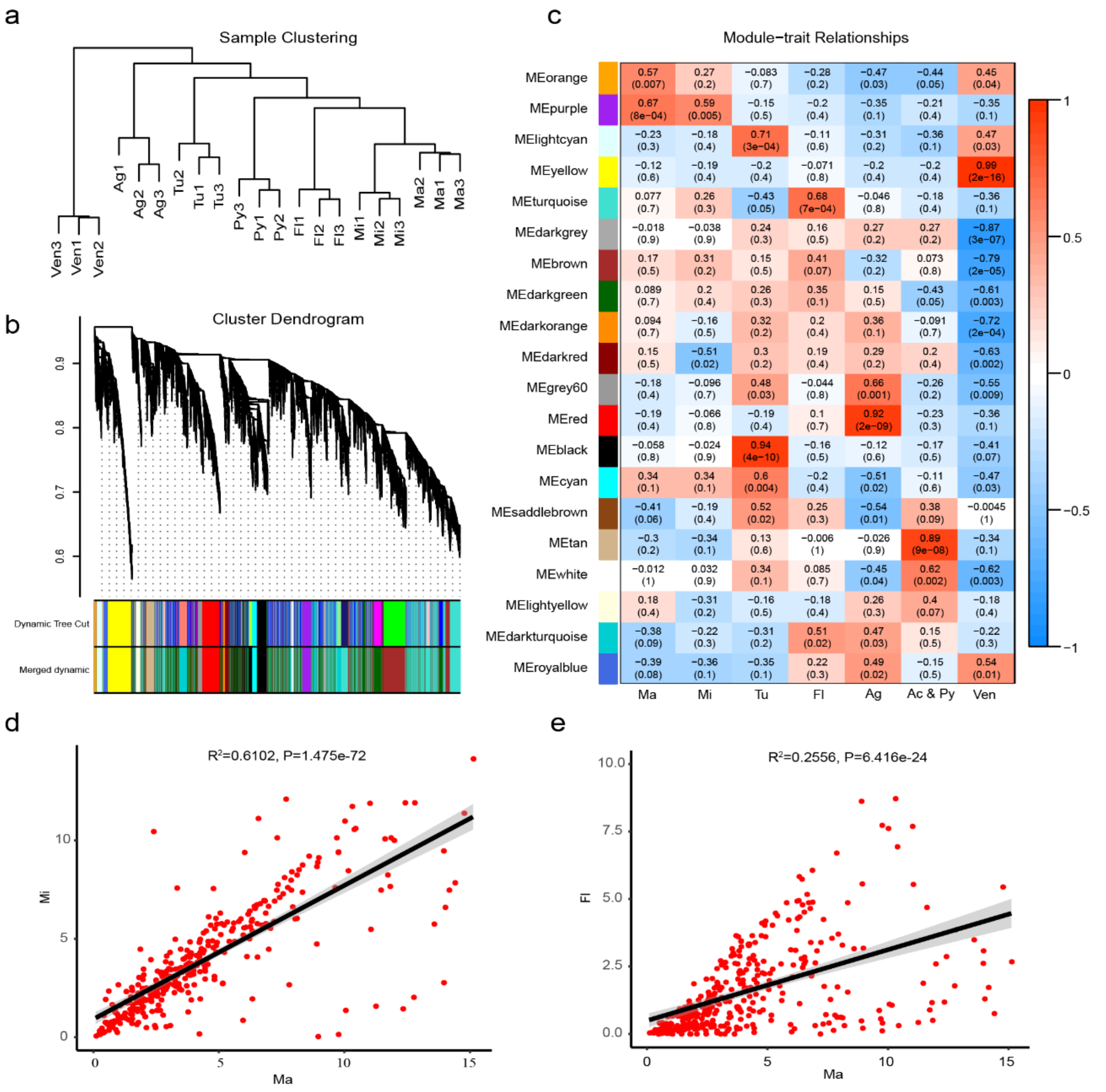

Supplementary Fig. 9: WGCNA of six silk glands and one venom gland. a, Expression clustering of gland samples. **b**, Cluster dendrogram of genes. Each color represented a module, 20 modules were obtained after merging. **c**, Module-trait relationships**. d**, **e**, Correlations of the genes of the purple module between Ma and Mi (**d**) or Fl (**e**). The coordinates represent the log_2(FPMK+1) values. R^2 indicates the square of Pearson's correlation coefficient. *P*-value assigned to each group was calculated by using two-tailed Student's t test. The shaded part indicates 95% confidence interval. The results indicated that Ma showed a closer relationship to Mi than to other silk gland tissues.

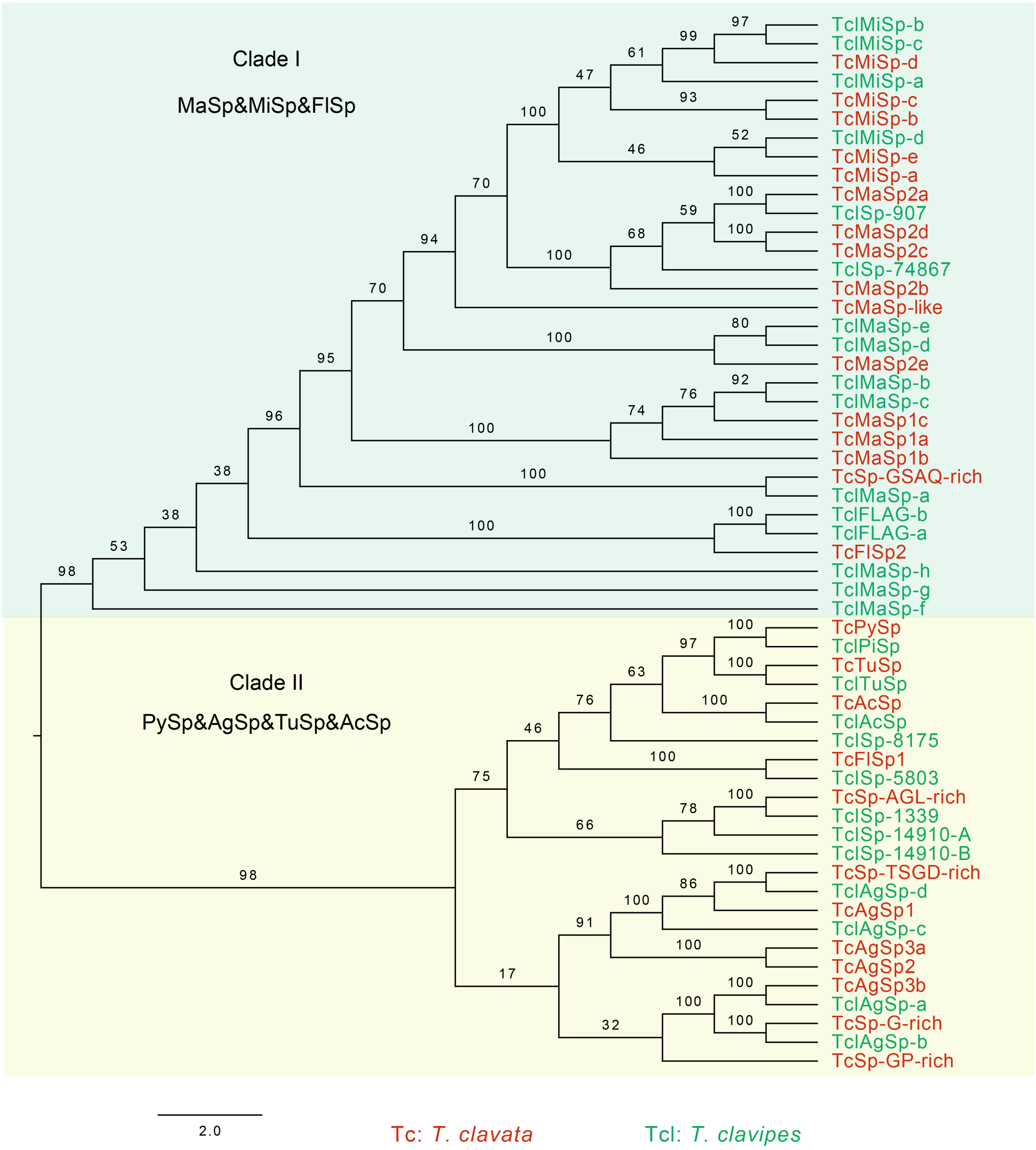

Supplementary Fig. 10: Phylogenetic relationships of 56 spidroins. The numbers on nodes represent the bootstrap values. FlSp and FLAG represent flagelliform gland spidroins, and PiSp and PySp represent pyriform gland spidroins.

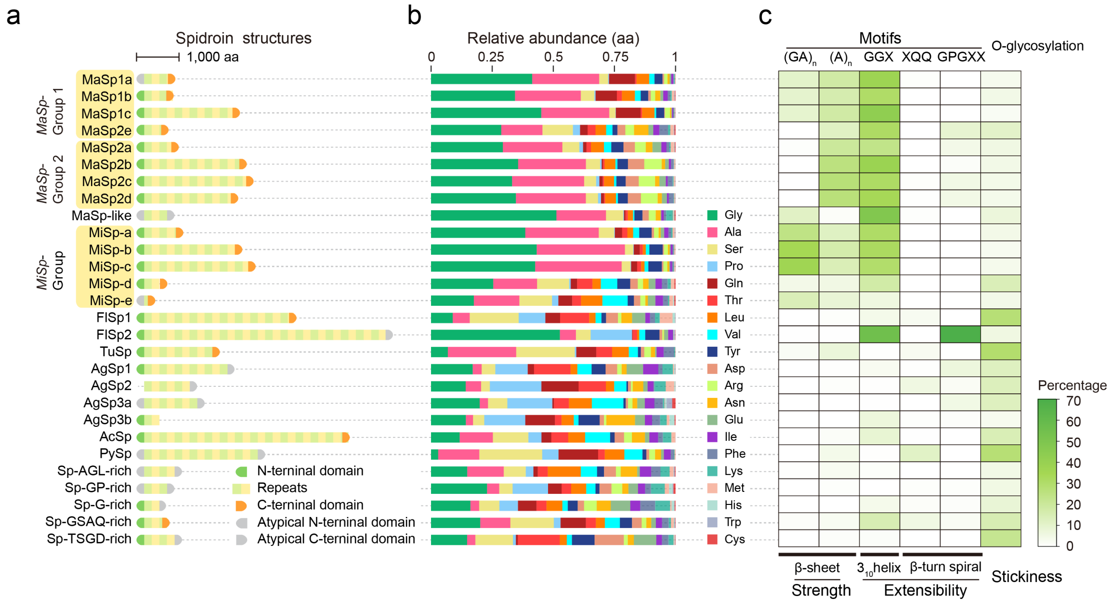

Supplementary Fig. 11: Spidroin sequence characteristics. a, Spidroin structure columns showing the N/C-terminal (green/orange box) and repeat domains. Each structure is drawn to scale. **b**, Amino acid contents of spidroins. **c**, Heatmap showing the variety of repetitive motifs in spidroins. The percentage represents the ratio of motif lengths/glycosylation sites to the total amino acid length of the corresponding spidroin.

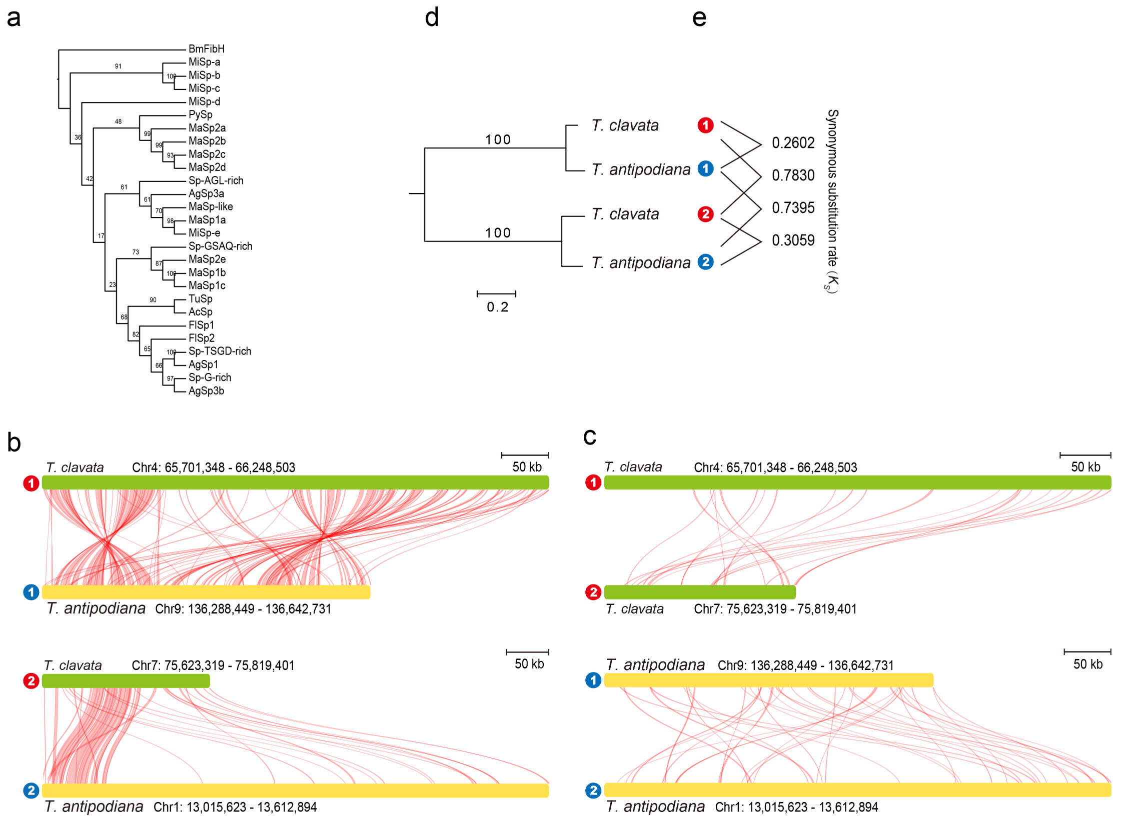

Supplementary Fig. 12: Spidroin evolution. **a**, Phylogenetic relationships of 28 spidroins and BmFibH (*B. mori*) as an outgroup. **b**, **c**, Collinearity of *MaSp*-Group 1 (**b**) and *MaSp*-Group 2 (**c**). The results showed that collinearity was higher between species than within species. **d**, **e**, The evolutionary relationships (**d**) and K_s values (**e**) of *MaSp* groups for *T. antipodiana* and *T. clavata*. The blue and red circles containing numbers represent the different *MaSp* groups of *T. antipodiana* and *T. clavata*, respectively. Left: the phylogenetic relationships within species were far stronger than those between species for *MaSp*-Group 1 and *MaSp*-Group 2. The numbers on nodes represent the bootstrap values. Right: the analysis of K_s values showed similar results (*Tc-MaSp*-Group 1 vs. *Ta-MaSp*-Group 1: 0.2602, which was less than the values of both *Tc-MaSp*-Group 1 vs. *Tc-MaSp*-Group 2: 0.7830 and *Ta-MaSp*-Group 1 vs. *Ta-MaSp*-Group 2: 0.7395).

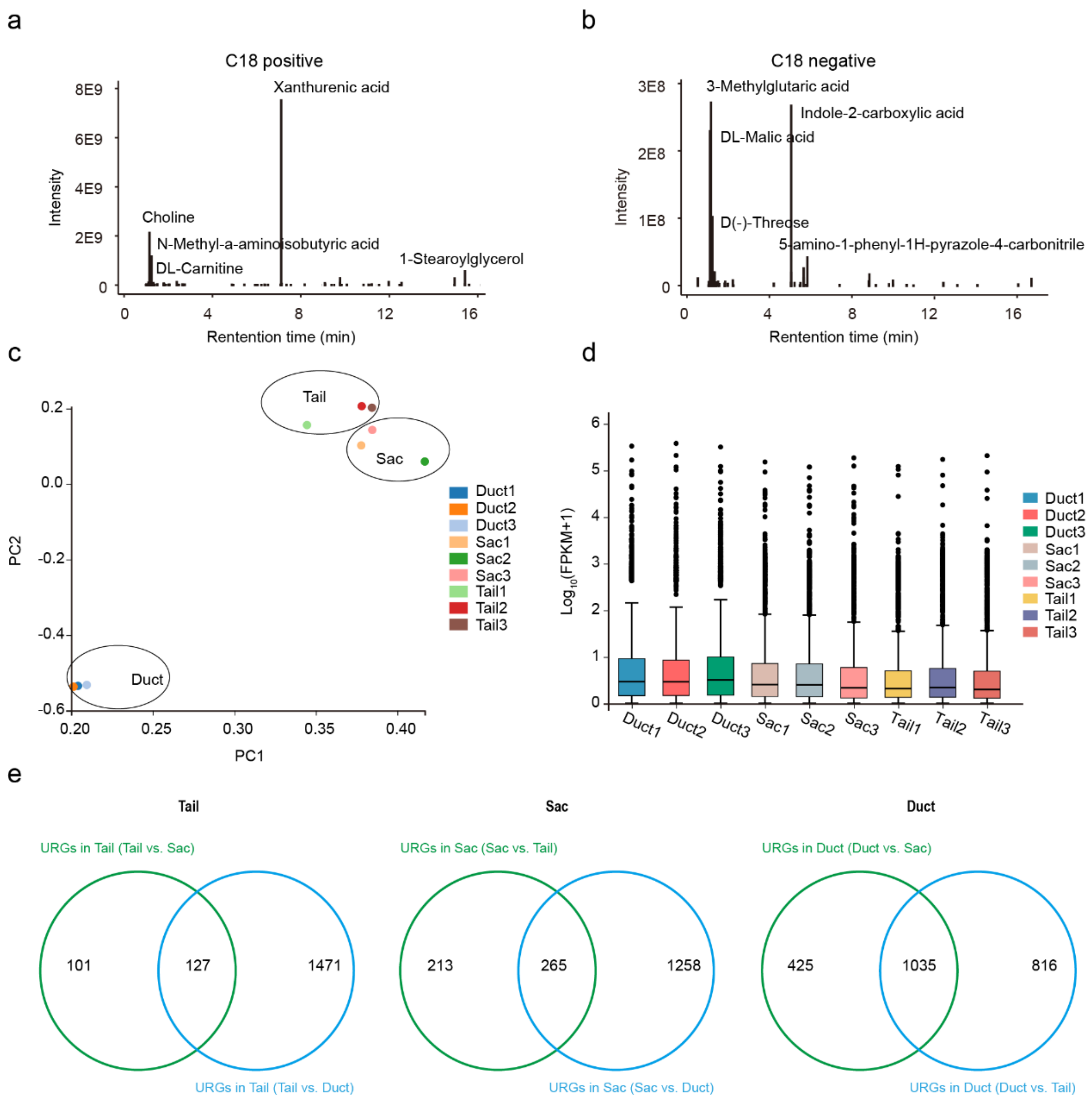

Supplementary Fig. 13: **a**, **b**, LC–MS analyses of the dragline silk metabolites under C18 positive (**a**) and negative (**b**) modes. The y axis represents the abundance of metabolites. **c**, Principal component analysis plot of the Tail, Sac, and Duct. (n = 3 independent experiments for each Ma segment). **d**, Boxplot of FPKM expression levels of the Tail, Sac, and Duct. (n = 3 independent experiments for each Ma segment). Date are presented as mean ± SD. Box plots show minimum to maximum (whiskers), 25–75% (box), median (band inside) with all data points. **e**, Flowchart of the screening of unique genes in the Tail, Sac, and Duct of the Ma gland.

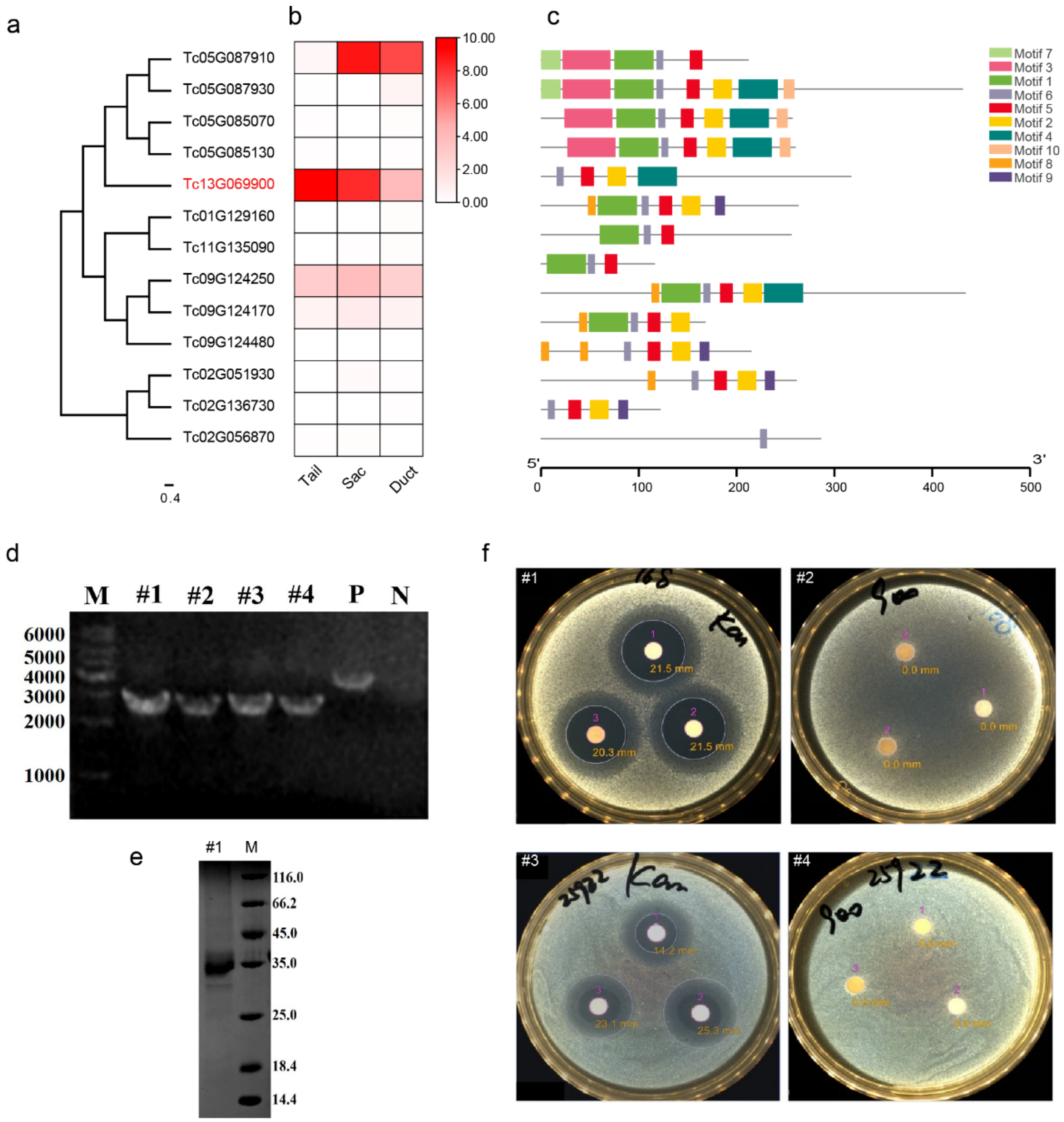

Supplementary Fig. 14: Venom protein function test. a, Phylogenetic tree of the CAP gene family to which Venom belongs. The protein marked red was used for functional study in Supplementary Fig. 14 d–f. **b**, Expression heatmap of 13 CAP genes in the Tail, Sac, and Duct. Expression values were normalized by log_2(FPKM+1). **c**, Conserved motif distribution. **d**, Agarose gel electrophoresis of the PCR product from the recombinant Bacmid plasmid (n = 4). M, maker. P, positive primer. N, negative primer. #1 - #4: recombinant bacmid strain of Venom protein. **e**, SDS–PAGE analyses of recombinant Venom protein. Similar results were obtained in two independent experiments and representative images are shown. **f**, Inhibition zone detection by the disk diffusion method. #1: Bacillus subtilis 168_Kanamycin (n = 3); #2: Bacillus subtilis 168_Venom (n = 3); #3: Escherichia coli ATCC 25922_Kanamycin (n = 3); #4: Escherichia coli ATCC 25922_Venom (n = 3). The results indicated that Venom had no bacteriostatic function against these two bacteria.

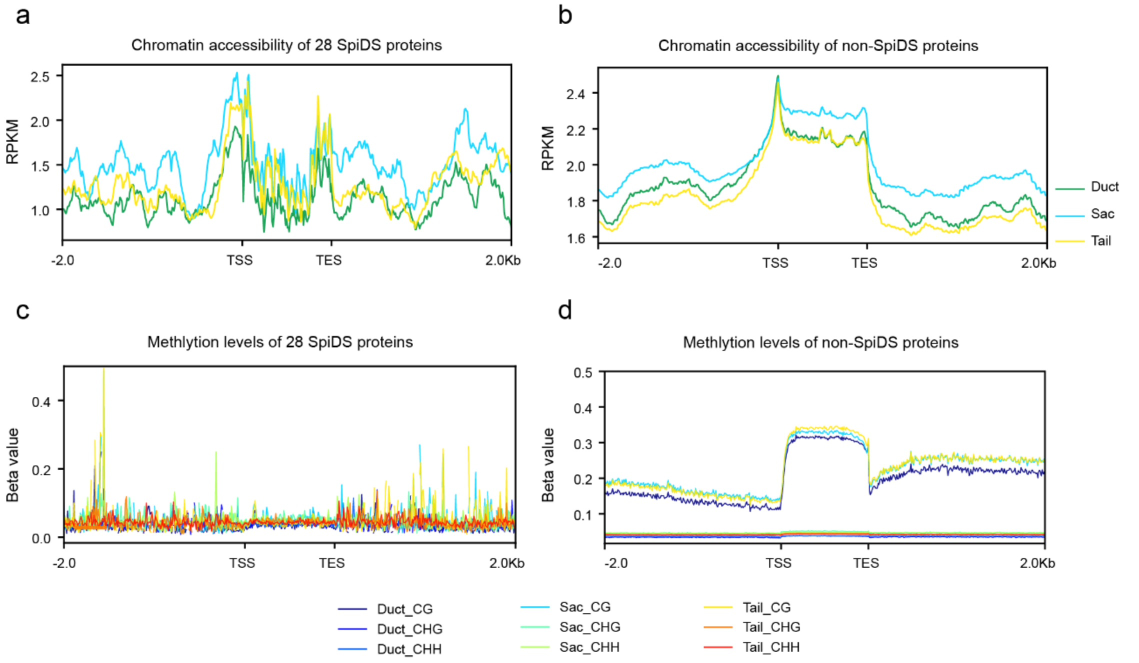

Supplementary Fig. 15: Chromatin accessibility and methylation levels of 28 spider dragline silk (SpiDS) and non-SpiDS genes. a, **b**, Chromatin accessibility of the 28 SpiDS (**a**) and non-SpiDS genes (**b**). The y axis represents the RPKM value calculated in the 50 bp window size. **c**, **d**, Methylation levels of the 28 SpiDS (**c**) and non-SpiDS (**d**) genes. The y axis represents the methylation level (beta value).

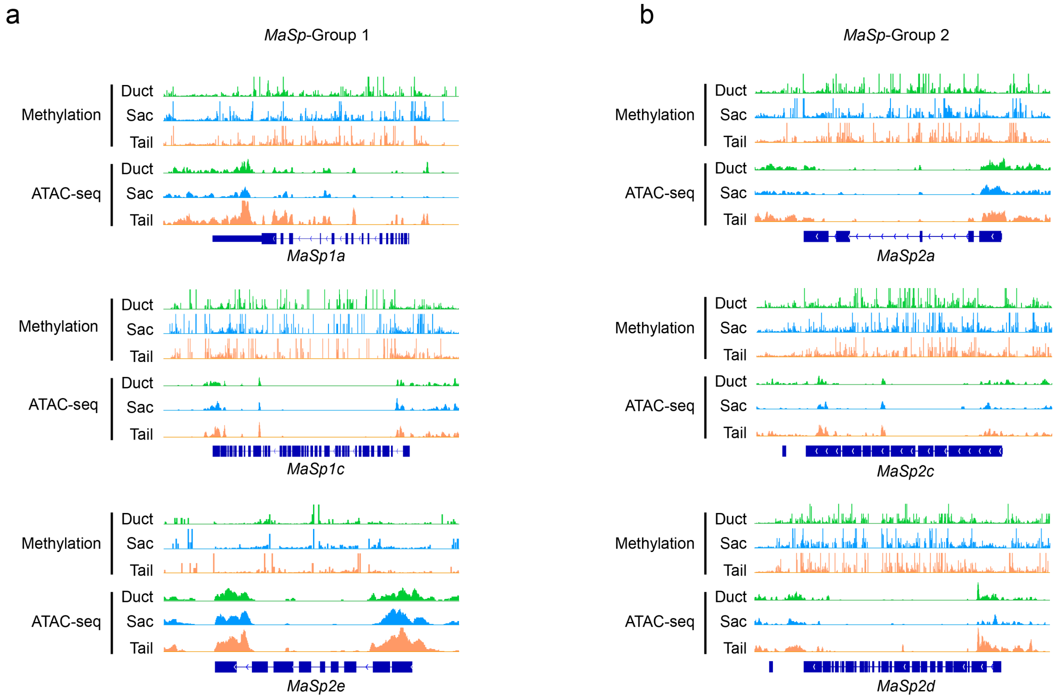

Supplementary Fig. 16: The methylation and chromatin accessibility peaks of *MaSp*-Group 1 (**a**) and *MaSp*-Group 2 (**b**). Different tissues are labelled with colored peaks. In gene structure diagrams, the blue wide boxes represent coding regions, the narrow boxes represent UTR regions, and the blue lines represent introns, the arrows represent gene orientation.

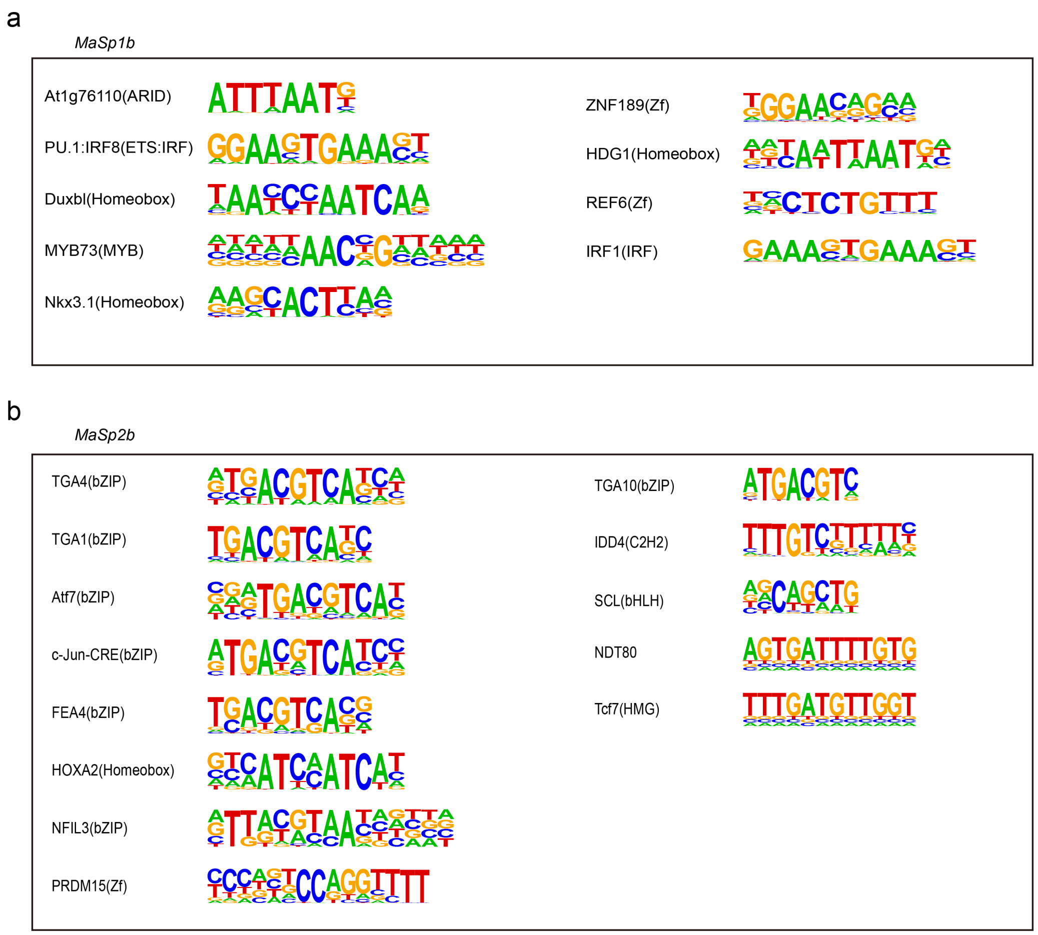

Supplementary Fig. 17: Specific motifs of *MaSp1b* and *MaSp2b*. a, Specific motifs of *MaSp1b*. **b**, Specific motifs of *MaSp2b*. *E*-value < $1e^{-10}$ was set as the criteria for screening significantly enriched motifs.

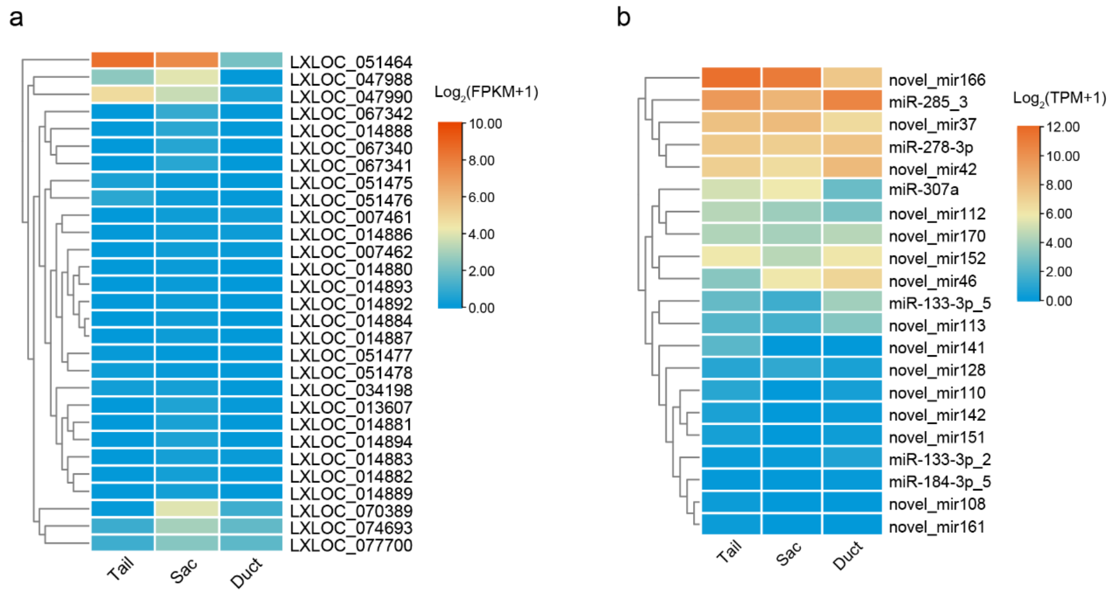

Supplementary Fig. 18: Expression heatmap of lncRNAs and miRNAs interacting with 28 SpiDS genes. a, Expression heatmap of lncRNAs in the Tail, Sac, and Duct. **b**, Expression heatmap of miRNAs in the Tail, Sac, and Duct.

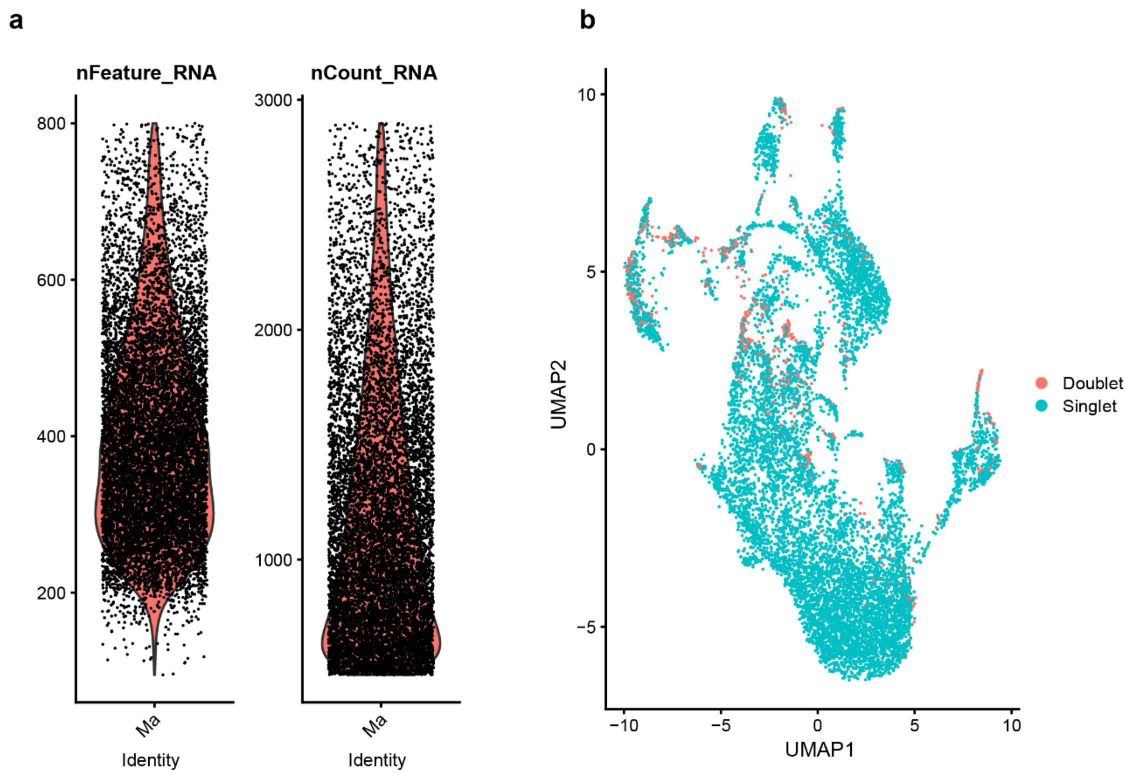

Supplementary Fig. 19: Ma gland scRNA-seq quality control. a, The number of expressed genes (left) and the number of UMIs (right) in each cell after quality control. **b**, Doublet cells were removed, and red dots represent possible doublet cells.

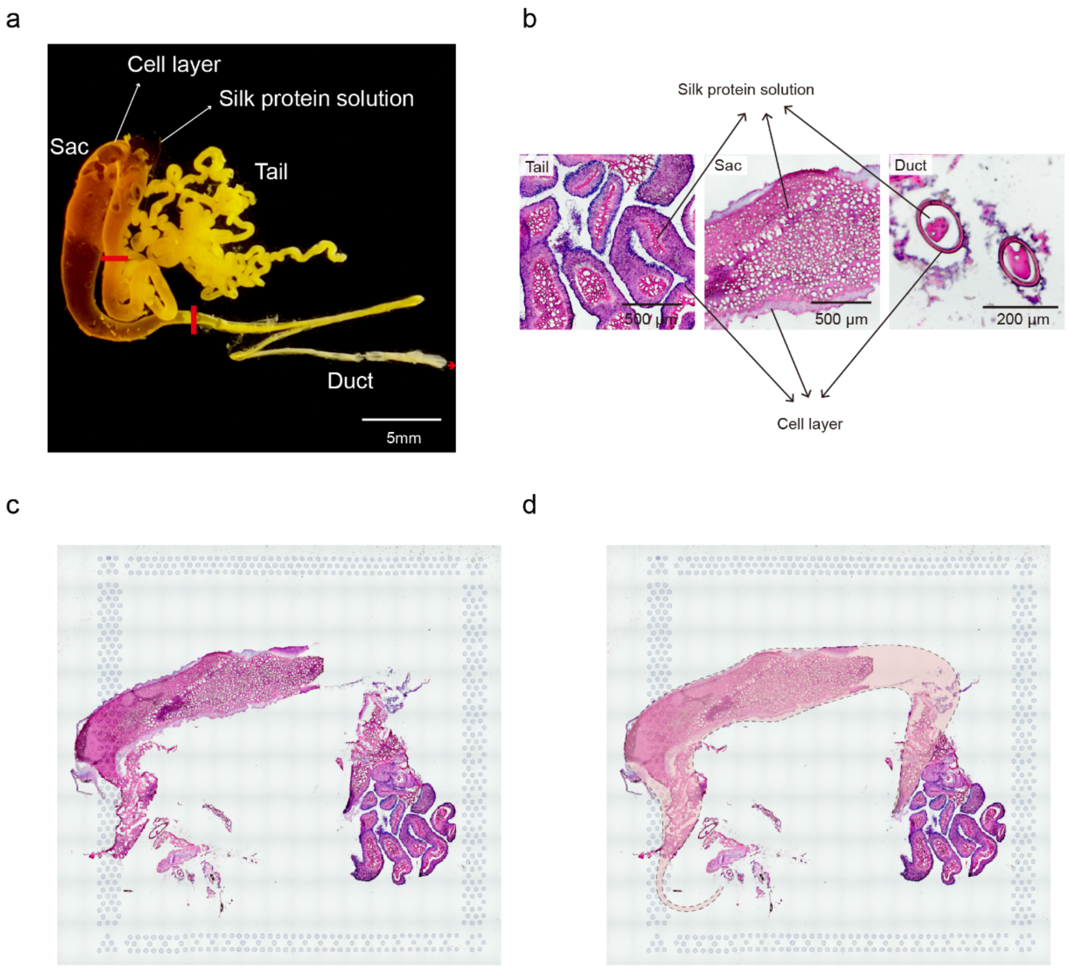

Supplementary Fig. 20: Morphological features and H.E. staining of Ma gland. a, Macroscopic appearance of the *T. clavata* Ma gland. The red arrow points to the flow direction of the silk protein solution. **b**, Detailed images of the Ma silk gland sections obtained by hematoxylin and eosin (H.E.) staining. Tail (longitudinal section), Sac (transverse section), and Duct (longitudinal section). **c**, H.E. staining of tissue sections for ST analysis. **d**, The outline of the Ma gland. The outline was depicted by dotted lines. Similar results were obtained in three independent experiments and representative images are shown for **a–d**.

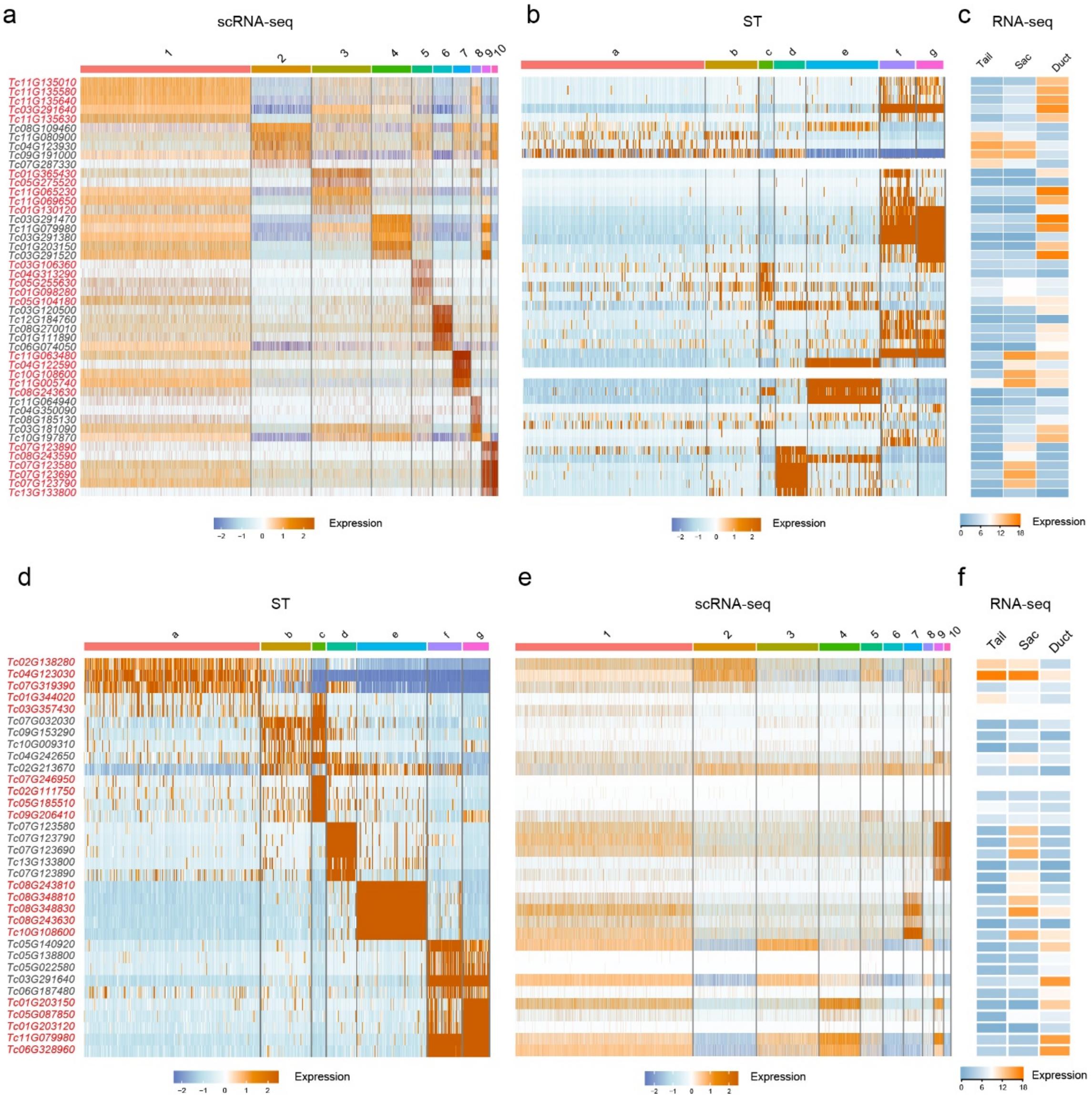

Supplementary Fig. 21: Expression heatmaps of marker genes. a–c, Expression heatmap of the top 5 single-cell marker genes based on scRNA-seq (**a**), ST (**b**), and bulk RNA-seq (**c**). **d–f**, Expression heatmap of the top 5 ST marker genes based on ST (**d**), scRNA-seq (**e**), and bulk RNA-seq (**f**).

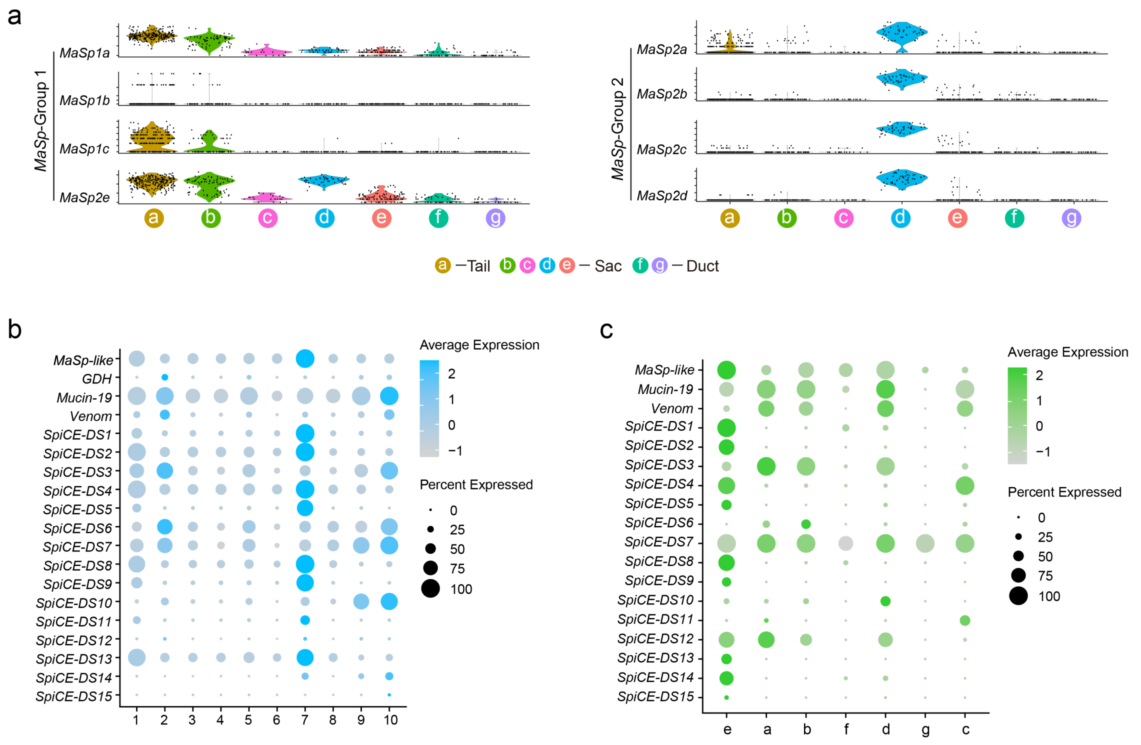

Supplementary Fig. 22: Expression patterns of SpiDS genes based on scRNA-seq and ST. a, Violin plots of *MaSp* gene expression across ST clusters. **b**, Expression bubble plots of *SpiDS* genes based on scRNA-Seq (*MiSp* was not expressed). **c**, Expression bubble plots of *SpiDS* genes based on ST (*MiSp* and *GDH* were not expressed).

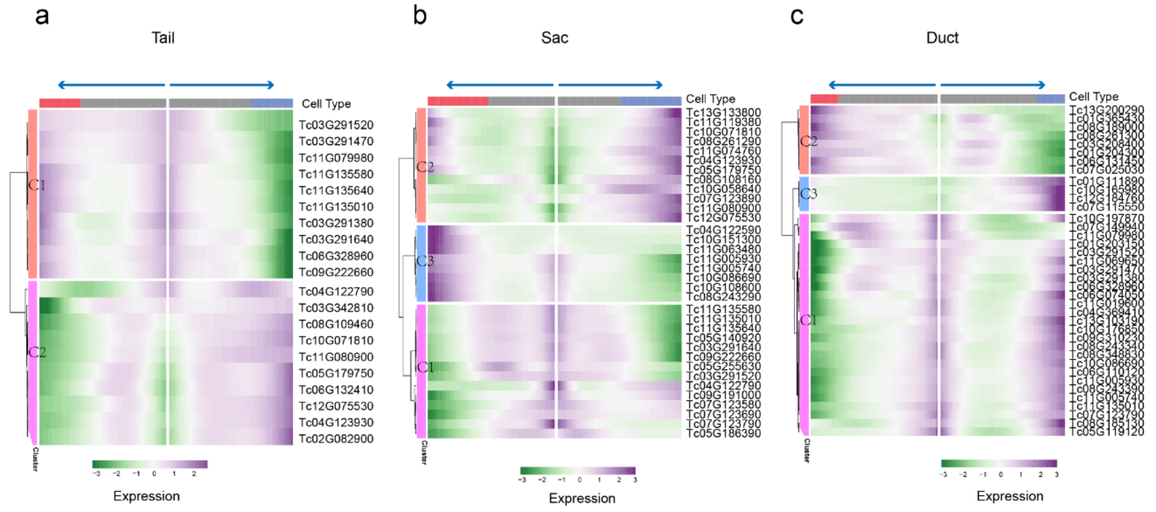

Supplementary Fig. 23: Developmental trajectories. a–**c**, Heatmap showing the expression of branch-dependent genes over pseudotime in the Tail (**a**), Sac (**b**), and Duct (**c**). Representative marker genes are indicated to the right of the heatmap. Both sides of the heatmap represent the end of the pseudotime period.

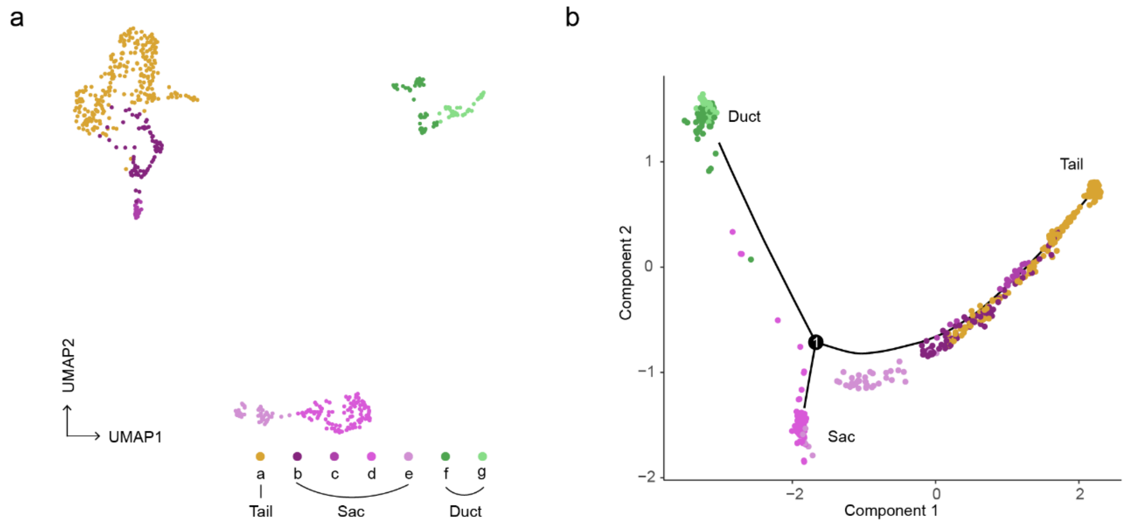

Supplementary Fig. 24: Spot clustering and developmental trajectory based on ST. a, UMAP visualization of seven putative clusters derived from 597 spots. **b**, Developmental trajectory of seven ST clusters.

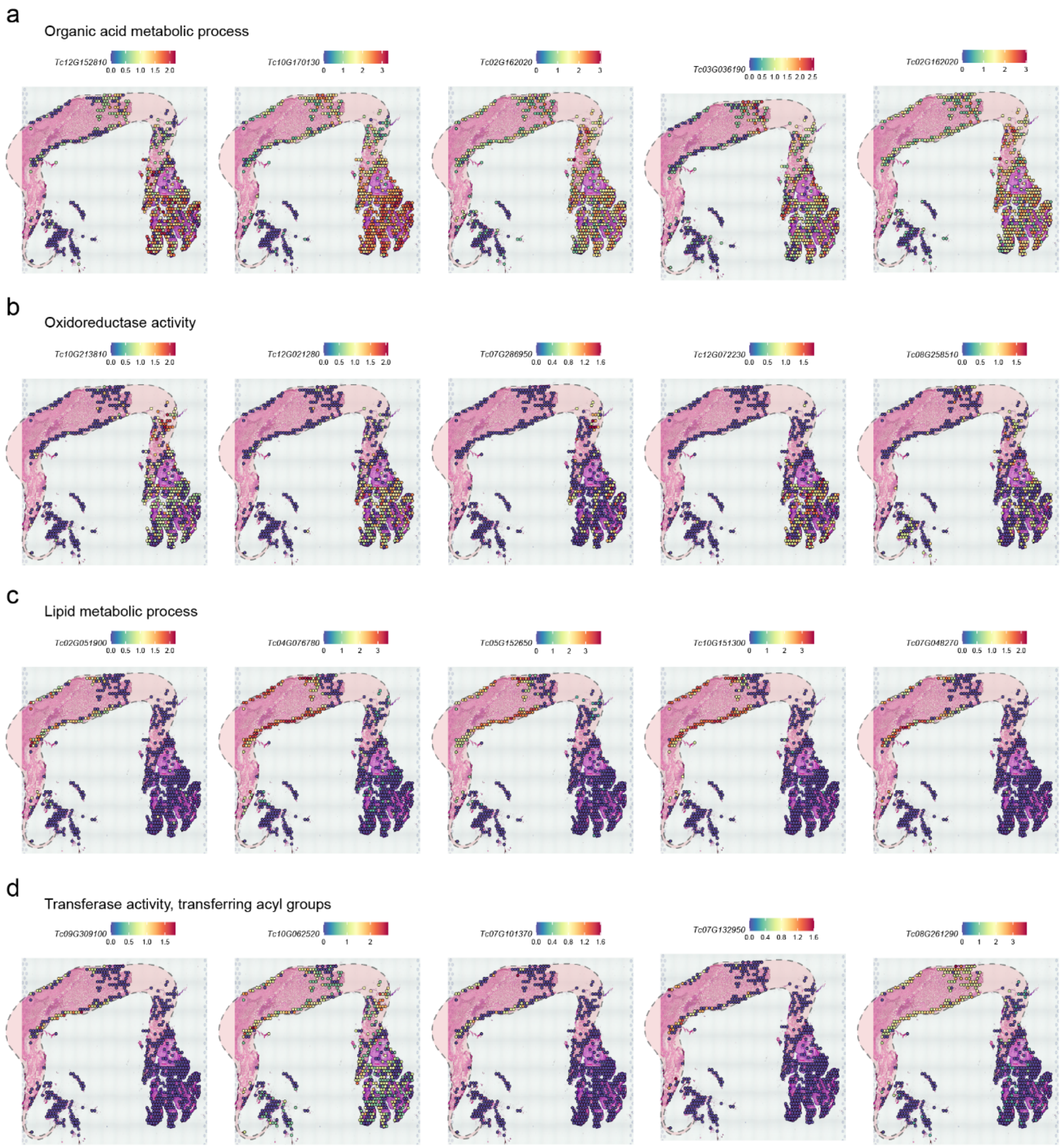

Supplementary Fig. 25: Feature plots of putative genes associated with dragline silk generation in the Tail and Sac based on ST. a, organic acid metabolic process. **b**, oxidoreductase activity. **c**, lipid metabolic process. **d**, transferase activity, transferring acyl groups.

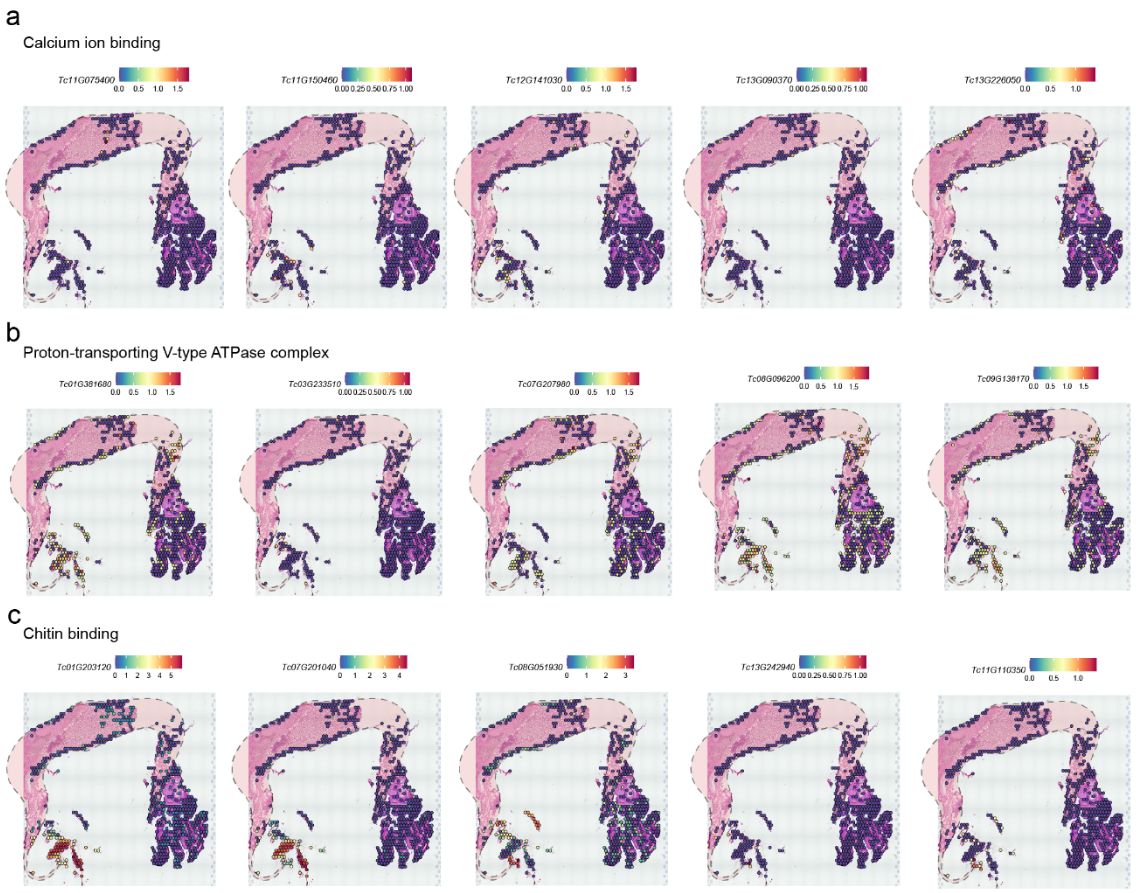

Supplementary Fig. 26: Feature plots of putative genes associated with dragline silk generation in the Duct based on ST. a, calcium ion binding. **b**, proton-transporting V-type ATPase complex. **c**, chitin binding.

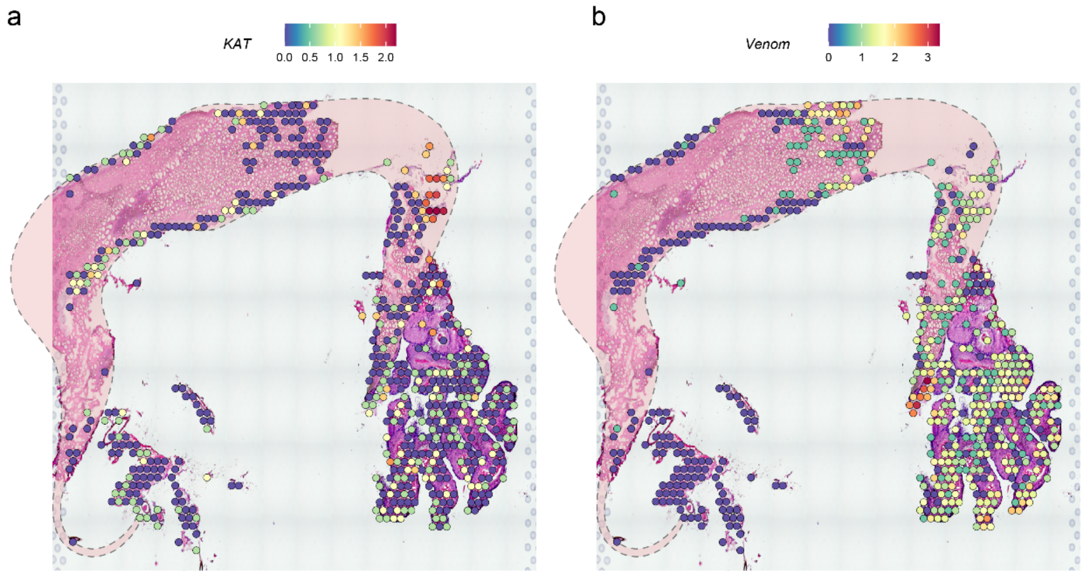

Supplementary Fig. 27: Feature plots of *KAT* and *Venom* based on ST.

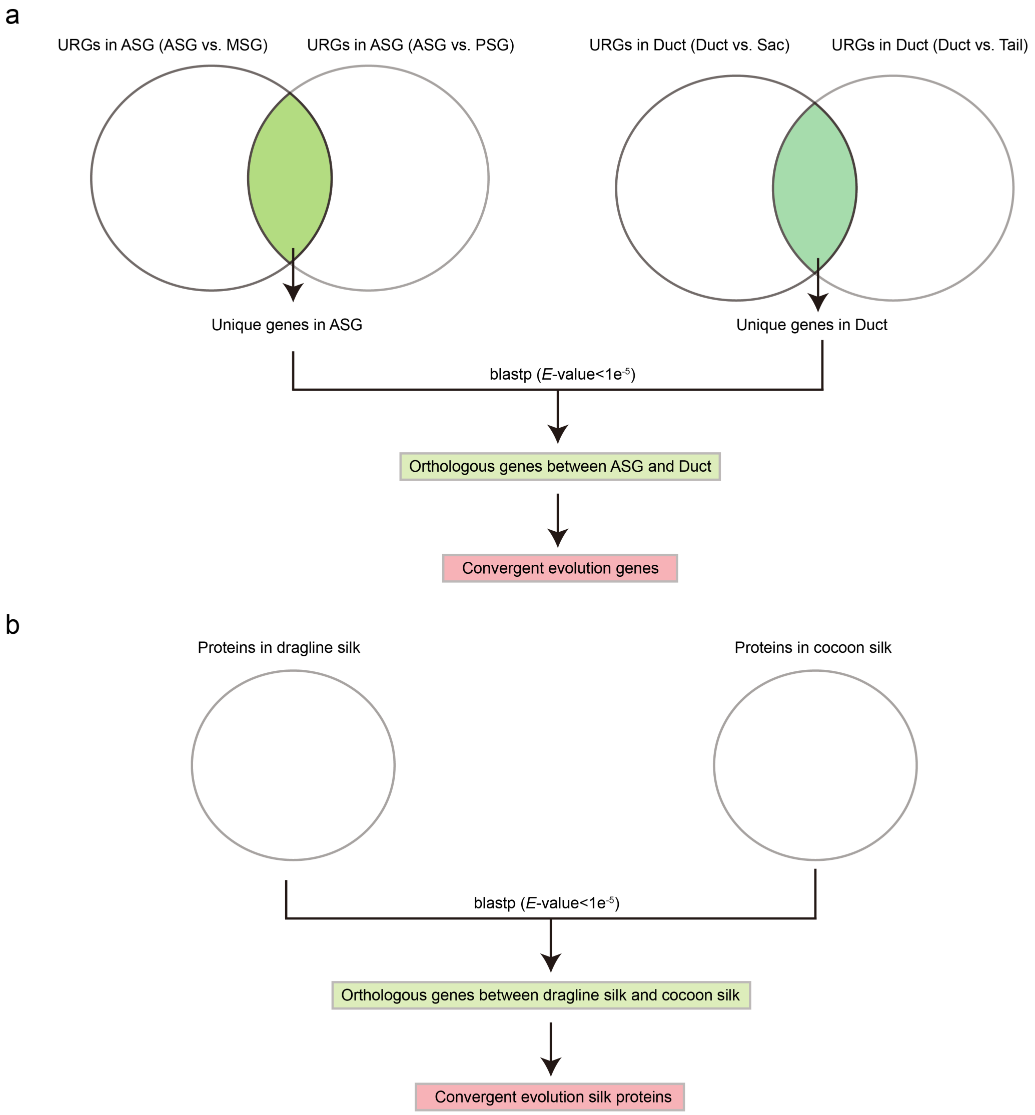

Supplementary Fig. 28: Pipeline of convergent evolution analysis. a, Flowchart of the screening of genes showing convergent evolution in the silk glands of *B. mori* (ASG) and *T. clavata* (Duct). The same methods were used for MSG vs. Sac and PSG vs. Tail. **b**, Flowchart of screening convergent silk proteins for dragline silk (*T. clavata*) and cocoon silk (*B. mori*).

	T. clavata	*B. mori*
Tail/PSG	Ma gland origin cell<br>*MaSp*-Group 1 synthesis cell	Fibroin protein synthesis cell<br>Death and remodeling regulation cell<br>Fibroin protein catabolism cell
Sac/MSG	Ma gland origin cell<br>*MaSp*-Group 1 synthesis cell<br>Ampullate lumen skeleton cell<br>Lipid synthesis cell<br>*MaSp*-Group 2 synthesis cell I<br>*MaSp*-Group 2 synthesis cell II	Sericin protein synthesis cell<br>Endoplasmic reticulum stress signaling cell<br>Sericin protein catabolism cell
Duct/ASG	Ma gland origin cell<br>Chitin synthesis cell<br>Unknown cell<br>Ion transport cell<br>pH adjustment cell	Liquid silk fibrosis cell<br>Epithelial cell remodeling cell<br>Chitin metabolism cell<br>Traction forces cell

Supplementary Fig. 29: Comparison of single cell types of spider (*T. clavata*) Ma gland and silkworm (*B. mori*) silk gland. There is a similar number of silk gland cell types between *T. clavata* and *B. mori*[64], but differentiated annotations except for the chitin-related process in the Duct/ASG.

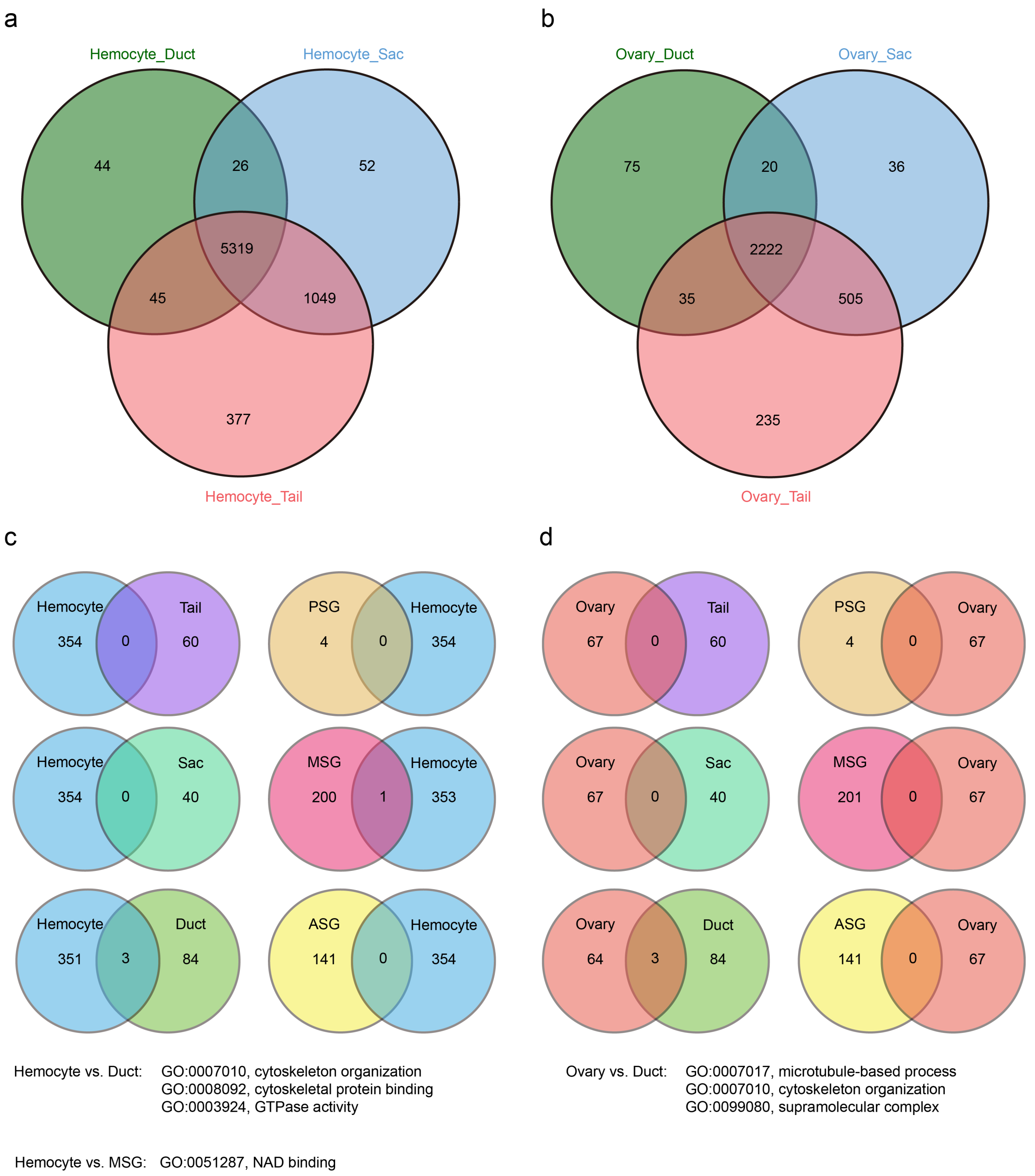

Supplemantary Fig. 30: **a**, **b**, Tissue-specific expressed gene analysis of the T. clavata hemocyte (**a**) and ovary (**b**). **c**, **d**, GO enrichment and Venn comparison analyses of hemocyte vs. silk gland (**c**) and ovary vs. silk gland (**d**). *P*-value < 0.05 was set as the criteria for screening enriched GO term.

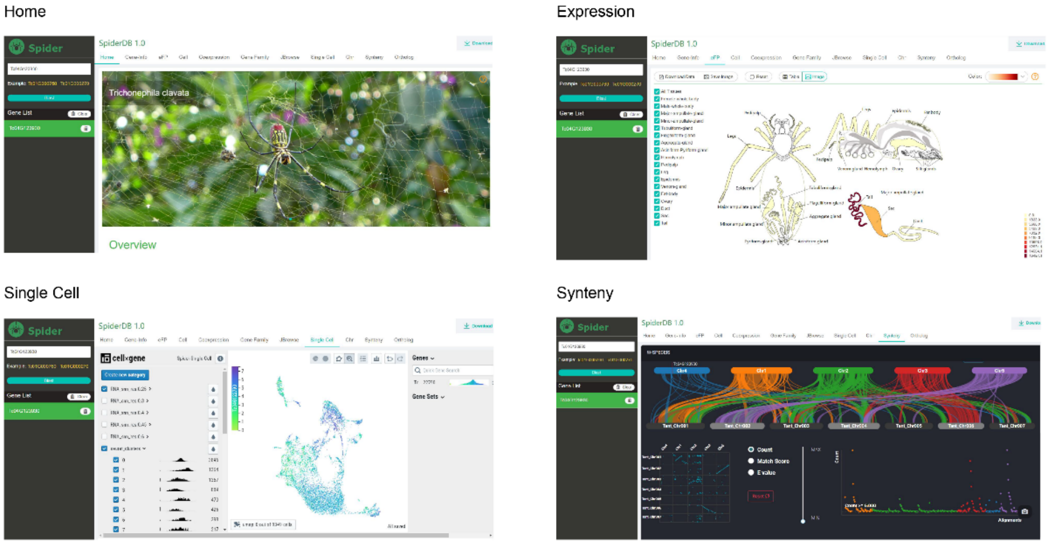

Supplementary Fig. 31: Several functional modules of SpiderDB. Home page, expression, single cell, synteny modules were listed, for more function modules refer to the spiderDB website (https://spider.bioinfotoolkits.net).

Supplementary note

Sample information, DNA sequencing, and genome survey

Sample information. *Trichonephila clavata* (basionym: *Nephila clavata,* Nephilidae, Trichonephila) is a golden orb-weaving spider with a wide distribution and high adaptability, and the silk of the female shows extremely high toughness and extraordinary rigidity[1-3]. In this study, *T. clavata* spiders were captured from the wild in Dali City, Yunnan Province, China.

DNA sequencing. Libraries for ONT and Illumina sequencing were constructed according to the platform manufacturers' instructions using high-quality genomic DNA extracted from specimens of adult female *T. clavata*. In total, ~349.95 Gb of Nanopore reads were generated, with the following characteristics: cleanData = 349.46 Gb, meanLength = 13,980 bp, and N50 = 19,639 bp (Supplementary Fig. 1a). A total of ~199.55 Gb of Illumina reads were also derived. The Hi-C library was constructed using the DpnII restriction endonuclease, and ~438.41 Gb of raw data were generated based on the Illumina HiSeq platform, with an insert size of 150 bp.

Genome survey. The genome size and heterozygosity ratio of *T. clavata* were estimated based on k-mer frequency distribution analysis using Illumina short reads with JellyFish[4] and GenomeScope[5] software. Under k-mer = 21, the estimated *T. clavata* genome size was approximately 2.72 Gb, and the heterozygosity ratio was 1.44% (Supplementary Fig. 1b; Supplementary Data 2).

Sex-chromosome analysis

The karyotype analysis (details as "Karyotyping") of *T. clavata* eggs revealed a chromosomal complement of 2n = 24 in males and 2n = 26 in females (X_1X_2 in males and $X_1X_1X_2X_2$ in females) (Fig. 1a). To identify the sex chromosomes of the *T. clavata* spiders, genomic DNA was extracted from five adult male and five adult female specimens for Pool-Seq library construction and sequencing, resulting in the generation of ~100.86 Gb and ~104.37 Gb of cleaned data from the female and male pools, respectively. BWA-MEM v0.7.17[6] was used to align the cleaned reads to the reference genome, and SAMtools rmdup[7] was used to remove the PCR duplicates. The fixation index (F_{ST}) and male- or female-specific SNPs of genomic position were calculated via PSASS (https://github.com/SexGenomicsToolkit/PSASS) with the following parameters: --window-size 10000 --output-resolution 1000. The results were visualized using the R package sgtr (https://github.com/SexGenomicsToolkit/sgtr). All R code is written in R studio 1.3.01093, the R Studio is an integrated development environment for R.

Because female *T. clavata* spiders had two more chromosomes (X_1 and X_2) than the male spiders, we calculated the read ratio per chromosome separately for females and males, where the read ratio per chromosome = the total number of reads on each chromosome/the total number of reads on all chromosomes. When males showed approximately half the read ratio for a particular chromosome relative to females, then the chromosome was considered a sex chromosome. Our analysis results indicated that Chr12 and Chr13 were sex chromosomes (Supplementary Fig. 2a). Some previous studies showed that the two smallest chromosomes were sex chromosomes by genome analysis[8,9], and our results accurately confirmed this conclusion. F_{ST} and male- or female-specific SNP analysis showed that there was no consistent trend across the female and male spiders (Supplementary Fig. 2b-d), but some high-density SNP sites were found in several regions of the genome.

Gene function annotation

Gene functions were predicted by aligning the protein sequences to the NCBI (https://www.ncbi.nlm.nih.gov/) NR database and the UniProt[10] (https://www.uniprot.org/UniProt/) database using the blastp[11] (E-value < 1e^{-5}) and the functional annotation of novel genes was performed via eggNOG-mapper[12] (http://eggnog-mapper.embl.de/) alignment with the eggNOG v5.0[13] (http://eggnog5.embl.de/) database. Pathway annotation and analysis were performed using the KEGG[14] (https://www.kegg.jp/) database.

Phylogenetic and divergence time analysis

The genome and GFF3 files of twelve species (*T. antipodiana*, *T. clavipes*, *A. ventricosus*, *A. bruennichi*, *L. hesperus*, *P. tepidariorum*, *S. dumicola*, *S. mimosarum*, *C. sculpturatus*, *T. gigas*, *H. longicornis*, *and S. maritima*) were downloaded from the NCBI (https://www.ncbi.nlm.nih.gov), GigaDB[15] (http://gigadb.org/), Dryad[16] (https://datadryad.org/stash) and NGDC[17] (https://ngdc.cncb.ac.cn/) databases (Supplementary Data 5). Protein-coding sequences were extracted using the gffread v0.12.7[18] tool, and the longest transcripts were retained. Single-copy orthologous protein sequences between species were identified by OrthoFinder v2.3.8[19]. Multiple sequence alignments were performed by using MUSCLE v3.8.31[20] and then concatenated to construct the species tree. The maximum likelihood (ML) tree was inferred using RAxML v8.2.12[21] (-x 12345 -# 1000 -m PROTGAMMALGX) with the optimal model identified by ModelFinder[22]. The tree was visualized in FigTree v1.4.4 (http://tree.bio.ed.ac.uk/software/figtree/). For divergence time, the aligned codon sequences were used to estimate divergence time by the MCMCtree (nsample = 20000, burnin = 2000) program of PAML v4.9[23] with three calibration points from the TimeTree[24] website (http://www.timetree.org/). One-to-one orthologous gene pairs were identified using the blastp[11] (E-value < 1e^{-5}) program based on protein comparison (all vs. all) between species. The aligned protein sequences were translated to codon sequences using Pal2nal v14[25], and the synonymous (K_s) substitution rates were calculated by using KaKs_Calculator 2.0[26] with the YN model.

We clustered the *T. clavata* gene models with the genes from the twelve other arthropod genomes and used 52 single-copy genes with one-to-one correspondence in the different genomes to reconstruct a phylogenetic tree. As a species of the Nephilidae family, *T. clavata* was most closely related to *T. antipodiana* among the species included in the analysis. The estimated divergence time between *T. clavata* and *T. antipodiana* was approximately 19.63 million years ago (Mya) (Supplementary Fig. 3a). The K_s values of orthologous genes from *T. clavata* vs. *T. antipodiana*, *T. clavata* vs. *T. clavipes*, *T. clavata* vs. *A. bruennichi*, *T. clavata* vs. *C. sculpturatus*, and *T. clavata* vs. *T. gigas* orthologous genes were calculated to be 0.06, 0.17, 1.6, 4.0, and 4.0, respectively (Supplementary Fig. 3b), indicating minimal divergence between *T. clavata* and *T. antipodiana*, consistent with the findings of the phylogenetic tree analysis.

Collinearity analysis

Collinear blocks were detected by using MCScanX[27] with all-to-all blastp[11] (E-value < 1e^{-5}) results (blocks with at least 5 pairs) within species. Accordingly, collinear blocks were identified between

species by using the JCVI (https://github.com/tanghaibao/jcvi) tool. Gene collinearity analysis was performed between the genomes of *T. antipodiana, A. bruennichi*, and *T. clavata*. The shared high collinearity revealed between *T. clavata* and *T. antipodiana* (Supplementary Fig. 4) exceeded that between *T. clavata* and *A. bruennichi*, which was consistent with the phylogenetic tree and divergence time results. These findings suggested that the collinearity between Nephilidae (*T. clavata*) and Araneidae (*A. bruennichi*) decreased after ancestral differentiation (~ 125.8 Mya) (Supplementary Fig. 3a), which may be related to the disruption of collinearity by the recent large-scale duplication of spider genes[28].

Demographic history analysis

The Bowtie2[29] tool was used to map the cleaned reads to the reference genome, and variable sites were identified using SAMtools v0.1.19[7] mpileup and BCFtools[30]. The effective population size was calculated using a mutation rate of $3.06 \times 10e^{-9}$ substitutions per site per year and a generation time of one year based on the pairwise sequentially Markovian coalescent (PSMC) (https://github.com/lh3/psmc). Mutation rates were estimated using the formula $\mu = K_s/2T$, where $K_s = 0.06$ and T = 19.63 Mya were employed as the synonymous substitution rates and the divergence time, respectively, between *T. clavata* and *T. antipodiana*.

The fluctuations of effective population size in each of the two lineages (*T. clavata* and *T. antipodiana*) were estimated by PSMC analysis. The results revealed a history of population fluctuations over nearly ten million years (Supplementary Fig. 5). One obvious population decline in both *T. clavata* and *T. antipodiana* near the Naynayxungla glaciation (NG: ~0.5 to 0.78 Mya) ice age; subsequently, the population size gradually decreased.

Repeat analysis

To assess TE content across species, we selected eight species (*T. clavata*, *A. bruennichi*, *P. tepidariorum*, *S. dumicola*, *C. sculpturatus*, *T. gigas, H. longicornis*, *and S. maritima*) with relatively high-quality genomes compared with other species. A custom repeat database was generated *de novo* with RepeatModeler v.2.0.2[31]. LTR_FINDER v1.06[32] was used to find long terminal repeat (LTR) sequences. Then, the repeat sequences were imported into RepeatMasker v 4.05[33] to search for TEs in the genome.

Among the eight species, *T. clavata, S. dumicola*, and *H. longicornis* showed relatively high repeat percentages of 53.94%, 56.3%, and 57.83%, respectively (Supplementary Data 4), and their genomes were also relatively large (Supplementary Fig. 6a). Among these repetitive sequences, the main repeat types in *T. clavata* were DNA transposons (22.9%) and LTR retrotransposons (14.41%), very similar to the results in *S. dumicola* (DNA transposons (19.60%) and LTR retrotransposons (20.18%)), whereas *H. longicornis* (longhorned tick) showed 19.74% LTR retrotransposons and 17.83% unclassified transposons. These results established that DNA transposons and LTR retrotransposons were responsible for the enlargement of the spider genome size.

Genome size analysis

To further confirm whether the genome size is determined mainly by DNA transposons and LTR retrotransposons in spiders, we analyzed *Hox* clusters in the same eight species (*T. clavata*, *A. bruennichi*,

P. tepidariorum, *S. dumicola*, *C. sculpturatus*, *T. gigas, H. longicornis*, *and S. maritima*) subjected to TE analysis via the methods described below. A total of 1,994 *Hox* protein sequences were downloaded from HomeoDB[34] (http://homeodb.zoo.ox.ac.uk/) to identify homologous *Hox* proteins based on analysis with the blastp[11] program (E-value < $1e^{-10}$), and we found that only two *Hox* clusters existed in each spider (Supplementary Fig. 6c). To better investigate the cause of genome size expansion, four spiders (*A. bruennichi*: 1.67 Gb, *T. clavata*: 2.62 Gb, *S. dumicola*: *2.55 Gb*, and *P. tepidariorum*: 1.44 Gb) were selected (because they have significant genome size differences) to evaluate the genome size by the above repeat annotation pipeline and visualize the different TE types using the IGV v2.9.4[35] tool. The divergence times of the LTRs were calculated by using LTR_retriever v2.9.0[36] with a mutation rate of $\mu = 3.06 \times 10e^{-9}$.

The comparisons of *T. clavata* (2.62 Gb) vs. *A. bruennichi* (1.67 Gb) and *S. dumicola* (2.55 Gb) vs. *P. tepidariorum* (1.44 Gb) indicated that the genome size expansion of *T. clavata and S. dumicola* was caused mainly by TE insertion. The most abundant TE types in the genome were DNA transposons (*T. clavata:* 22.9% vs. *A. bruennichi:* 11.98%, *S. dumicola*: 19.6% vs. *P. tepidariorum:* 7.44%) and LTR retrotransposons (*T. clavata:* 14.41% vs. *A. bruennichi:* 4.21%, *S. dumicola*: 20.18% vs. *P. tepidariorum:* 0.55%) (Supplementary Data 4; Supplementary Fig. 6d). When we more closely examined the distances between different genes of the *Hox* gene clusters, it was found that intergenic/intronic elongation had occurred independently in *T. clavata*, caused by the multiplication of different types of TEs (Supplementary Fig. 7a,b). We further analyzed the insertion times of LTRs and found that LTR insertion was a continuous process that had occurred within the last ten million years in *T. clavata* and *S. dumicola* (Supplementary Fig. 7c,d). This is much more recent than the divergence time of either *T. clavata* or *S. dumicola* (Supplementary Fig. 3a), implying that LTRs had expanded independently within species. Our results further highlight that the main drivers of spider genome size expansion are DNA transposon and LTR retrotransposon expansion.

Gene family expansion and contraction analysis

The above eight species were also used to assess gene family expansion and contraction. After the phylogenetic tree and divergence times were analyzed according to the above steps (see the "Phylogenetic and divergence time analysis" section), Café v4.2.1[37] software was used to identify the numbers of gene families that had undergone expansions or contractions between species (retaining gene families with at least one and no more than 200 members). Gene Ontology (GO) enrichment analysis was performed in clusterProfiler v4.2.1[38] and visualized via the REVIGO[39] tool.

Among 6,913 identified orthologous gene families (Supplementary Fig. 6b), we found that 5,178 gene families had experienced losses in *T. clavata*, while 700, 942, and 93 families underwent expansion, contraction, and rapid evolution, respectively. The GO enrichment analysis showed that the expanded gene families were mainly enriched in the following GO terms: in the biological process (BP) category (Supplementary Fig. 8a), steroid metabolic process (GO:0008202), hormone metabolic process (GO:0042445), and organic hydroxy compound metabolic process (GO:1901615); in the cellular component (CC) category (Supplementary Fig. 8b), endoplasmic reticulum membrane (GO:0005789) and extracellular matrix (GO:0031012); and in the molecular function (MF) category (Supplementary Fig. 8c), monooxygenase activity (GO:0004497), steroid binding (GO:0005496) and demethylase

activity (GO:0032451). The contracted gene families were mainly enriched in the following GO terms: in the BP category (Supplementary Fig. 8d), synaptic transmission, GABAergic (GO:0051932), regulation of gene expression, epigenetic (GO:0040029), and adult somatic muscle development (GO:0007527), and in the CC category (Supplementary Fig. 8e), fusome (GO:0045169) and distal axon (GO:0150034).

Coexpression analysis

Fragments Per Kilobase of exon model per Million mapped fragments (FPKM) values representing expression levels were calculated as follows: 1) the raw files of 18 tissues were cleaned by using fastp v0.23.2[40] software; 2) the HISAT v2.2.1[41] tool was used to map the cleaned reads to the reference genome; and 3) FPKM values were calculated by using StringTie v2.1.5[41]. To assess expression correlations between silk gland tissues, the data from seven tissues (Ma, Mi, Fl, Tu, Ag, Ac&Py, and Ven glands) were clustered according to the hierarchical clustering (HC) method, and coexpression network relationships between genes were identified through weighted gene coexpression network analysis with the WGCNA[42] package.

The clustering of samples from the seven tissues is shown in Supplementary Fig. 9a. The Ma and Mi glands showed the highest correlations of gene expression, followed by the Fl gland. Using WGCNA, 20 coexpression modules were constructed based on the 37,607 genes from the 21 samples of seven *T. clavata* tissues (Supplementary Fig. 9a,b), in which the MEpurple module was significantly positively associated with the Ma and Mi glands but was not associated with other glands (Supplementary Fig. 9c). Meanwhile, the scatter plot of the 217 genes of the MEpurple module also generated consistent results showing that the Ma gland was more highly correlated with the Mi gland (Supplementary Fig. 9d,e).

Phylogenetic analysis of spidroins

A total of 28 putative spidroins were identified via the methods described in the "Materials and Methods" section of the main text (under "Spidroin analysis") and classified according to motif sequence types and tissue expression levels. To name these proteins, we estimated the phylogenetic tree of the 28 spidroin proteins with BmFibH as the outgroup (Supplementary Fig. 12a) by using IQ-TREE[43] (-m PMB+F+R4 -bb 1000 -bnni) software. We also constructed a phylogenetic tree of *T. clavata and T. clavipes* spidroins and found that these spidroins were clustered into two large clades (clade I and clade II) (Supplementary Fig. 10). Clade I included all MaSp, MiSp, and FlSp members, and clade II included all PySp, AgSp, TuSp, and AcSp members. Our multiple findings established the high similarity of Ma, Mi, and Fl glands based on morphology (Fig. 1g) and molecular evidence (Supplementary Fig. 9a and 10).

Spidroin sequence analysis

Amino acid contents and motifs were quantified using a Perl script. The motifs included (GA)n, (A)n, GGX, XQQ, and GPGXX. O-glycosylation was predicted with the NetOGlyc v4.0[44] tool. In *T. clavata*, the lengths of the spidroin coding sequences showed considerable diversity, with the number of encoded amino acids ranging from 194 (MiSp–e) to 7,819 (FlSp2) (Supplementary Fig. 11a). The analysis of spidroin amino acid contents showed that glycine (G, 29.0%), alanine (A, 18.4%), serine (S, 10.3%),

proline (P, 6.8%), and glutamine (Q, 4.5%) were the five most abundant residues (Supplementary Fig. 11b), contributing to the physical properties of silk fibers[45]. We therefore investigated the variety of typical repeat motifs present in spidroins, which included β-sheet motifs ((GA)n and (A)n), 3_{10} helix motifs (GGX), β-turn spiral motifs (XQQ and GPGXX), putative O-glycosylation sites, and many long and regular motif sequences (Supplementary Data 8). Notably, the β-sheet and 3_{10}-helix motifs frequently occurred in MaSps and MiSps, and β-turn spiral motifs and O-glycosylation sites frequently occurred in the rest of the spidroins (Supplementary Fig. 11c; Supplementary Data 9), suggesting Ma and Mi silk with high strength/extensibility and other silk with high extensibility/stickiness. These results indicated that the variation in motif assembly resulted in the differentiation of the spidroin structure, thus conferring the different mechanical properties of silks.

MaSp **group identification and evolutionary analysis**

TBtools[46] was used to visualize the positions of spidroin genes on chromosomes based on the location information of the gff3 file. We found that the spidroin genes were located on nine of the 13 *T. clavata* chromosomes and that the *MaSp1a–c* & *MaSp2e*, *MaSp2a–d*, and *MiSp-a–e* genes were distributed in three independent areas (Fig 1c,d). Interestingly, we noted that the genomic loci of *MaSp1a–c* & *MaSp2e*, *MaSp2a–d*, and *MiSp-a–e* were located in tandem or close to each other, so we named them *Tc-MaSp*-Group 1, *Tc-MaSp*-Group 2, and *Tc-MiSp*-Group, respectively. Additionally, to assess whether the spidroin groups also existed in other species, we searched for the closely related species *T. antipodean* spider, and the analysis results indicated that the *Ta-MaSp*-Group 1, *Ta-MaSp*-Group 2, and *Ta-MiSp*-Group also existed in *T. antipodiana*. Recently, spidroin clustering has also been reported in a black widow spider (*L. elegans*)[47]. The results indicated that these spidroin groups or clusters existed in some spider genomes.

To further explore the evolving relationship between *MaSp* groups. The RectChr (https://github.com/BGI-shenzhen/RectChr/) tool was used to calculate and visualize the collinearity between *MaSp* groups. K_s was calculated by KaKs_Calculator 2.0[26] using the YN model based on the concatenated and aligned nucleic acid sequences. In *MaSp*-Group 1 or *MaSp*-Group 2, an obvious collinear relationship was observed between *T. clavata* and *T. antipodiana* (Supplementary Fig. 12b,c), whereas weaker collinearity was observed within species, implying closer affinities of *MaSp*-Groups between species. Likewise, the phylogenetic analysis also strongly supported (=100) the sister relationship of *Tc-MaSp*-Group 1 and *Ta-MaSp*-Group 1, as well as that of *Tc-MaSp*-Group 2 and *Ta-MaSp*-Group 2 (Supplementary Fig. 12d). The K_s analysis revealed that the K_s values between species were lower than those within species (Supplementary Fig. 12e): the value for *Tc-MaSp*-Group 1 vs. *Ta-MaSp*-Group 1 was 0.2602, while that for *Tc-MaSp*-Group 2 vs. *Ta-MaSp*-Group 2 was 0.3059, which was greater than the shared K_s peak (K_s =0.06, Supplementary Fig. 3b) between *T. clavata* and *T. antipodiana*. However, the value for *Tc-MaSp*-Group 1 vs. *Tc-MaSp*-Group 2 was 0.7830, and that for *Ta-MaSp*-Group 1 vs. *Ta-MaSp*-Group 2 was 0.7395, which are lower than the value of the shared K_s peak between *T. clavata* and *A. bruennichi* (K_s =1.6). These findings showed that the orthologous *MaSp* groups between species were more closely related than those within species, although more high-quality genomic data are needed to validate this finding.

Identification of the golden pigment of dragline silk

Metabolome analysis was performed via the methods described in the "Materials and Methods" section of the text (details in the "Metabolomics" subsection), and a total of 180 metabolites were identified in dragline silk (Supplementary Data 12). Some previous studies have indicated that the golden pigments of spider silk were quinones (mostly benzoquinone and naphthoquinone) or xanthurenic acid (XA)[48,49]. Here, the five most abundant metabolites included XA, choline, N-methyl-a-aminoisobutyric acid, 1-stearoylglycerol, and DL-carnitine (Supplementary Fig. 13a,b). To confirm the identity of the golden pigment, the dragline silk of *T. clavata* was cut into pieces and dissolved in 200 µL of methanol solution, and the XA ($C_{10}H_7NO_4$) standard was dissolved in 200 µL of acetonitrile solution. Then, LC–MS was used to determine whether XA was present in the silk solution. LC–MS analyses were performed on a Waters ACQUITY H-Class UPLC–MS system coupled to a photodiode array detector and an SQD2 MS detector with an ESI source. Chromatographic separation was performed on a C18 column (ACQUITY UPLC BEH, 1.7 mm, 2.1 mm × 100 mm, Waters) at 35 °C. As shown in Fig. 2e, XA was the major pigment of the silk; this result was also confirmed by another recent study[50]. Hence, we then focused on the synthesis pathway of XA and related genes (see the text "Dragline silk origin and the functional character of the tri-section Ma gland" subsection for details), the results showed that XA may be synthesized in Tail, Sac, and Duct.

Ma gland transcriptomic analysis

The Ma gland consists of three distinct parts, the Tail, Sac, and Duct (Fig. 2a and 5a), and these segments were examined by transcriptome sequencing. Correlations were calculated between samples based on Pearson's correlation coefficients in R (cor function). Differentially expressed genes (DEGs) were examined using the DESeq2[51] package. Sample correlation clustering analysis (Fig. 2f), expression clustering (Fig. 2g), and principal component analysis (Supplementary Fig. 13c) both showed some similar features between the Tail and Sac. To identify specific genes in the Tail, Sac, and Duct of the Ma gland, we used the strategy shown in Supplementary Fig. 13e to screen out specific genes in each tissue. Here, the overlapping upregulated differentially expressed genes (URGs) in Tail vs. Sac and Tail vs. Duct were considered to be unique to Tail, and the same analysis was also performed for Sac and Duct. A total of 127, 265, and 1035 unique genes were identified in the Tail, Sac, and Duct, respectively. GO enrichment analysis was performed using the clusterProfiler[38] package in R, and the terms with a Q-value ≤ 0.01 were regarded as significantly enriched (Supplementary Data 14).

Exploring the function of the dragline silk venom protein

Within dragline silk proteomic results, we found a Venom protein (Tc13G069900; we named it Venom) that was also expressed in the Ma (FPKM, Duct: 15.20, Sac: 312.60, Tail: 706.88) gland (Fig. 2h, Supplementary Data 10). Functional annotation showed that the protein belonged to the cysteine-rich secretory protein, antigen 5, and pathogenesis-related protein 1 (CAP) superfamily based on analysis with the SMART[52] online tool (http://smart.embl-heidelberg.de/). Then, we identified all members of the CAP family in *T. clavata* and constructed a phylogenetic tree (Supplementary Fig. 14a, the tree was constructed by MEGAX[53]), gene expression heatmap (Supplementary Fig. 14b), and domain structure (Supplementary Fig. 14c) analyses. The domain structure was analyzed by the MEME[54] online tool

(https://meme-suite.org/meme/tools/meme). We found that two genes (Tc13G069900 and Tc05G087910) were highly expressed in the Ma gland, while the Venom (Tc13G069900) with an incomplete motif structure (Supplementary Fig. 14c) was also exists on dragline silk. We hypothesized that this protein might not play a role in toxicity and that it might have other undiscovered potential functions.

Based on the above findings, we next performed an antibacterial activity experiment. Bacmid vector construction: the recombinant Bacmid plasmid was constructed with the Bac-to-Bac expression system. Specific primers (Tc13G069900F: ACCGTCCCACCATCGGGCGCGGATCCATGCACCACCACCACCATCACCAGAACATCGCC, Tc13G069900R: GCTCGTCGACGTAGGCCTTTGAATTCGTTCTGGCAGCTGCCGTCGACAGCCAGGATCTT) and universal primers (M13-F: GTTTTCCCAGTCACGAC, M13-R: CAGGAAACAGCTATGAC) were employed for PCR identification. The PCR products were stained after agarose gel electrophoresis, and the results showed that the correct size of the recombinant bacmid fragment (Supplementary Fig. 14d). Protein purification: a total of 100 μg of Venom protein was obtained, purified, and concentrated by Ni-NTA purification technology. The purity was greater than 85%, and the concentration was 0.5 mg/mL. Protein antibacterial test: using the disk diffusion test to conduct the antibacterial experiment, we selected Gram-positive bacterium (*Bacillus subtilis*) and Gram-negative bacterium (*Escherichia coli*) for testing, and kanamycin was used as the control group. The size of the bacterial inhibition zone was analyzed, and no change in the zone size was observed (Supplementary Fig. 14f). This result indicated that Venom had no bacteriostatic function in these two bacteria; thus, it may have other potential functions.

Chromatin accessibility and methylation level analysis

To assess the relationship between chromatin accessibility and methylation and whether they potentially play a joint role in coregulating spider dragline silk (SpiDS) gene expression, we compared the distributions of chromatin accessibility and methylation levels for Tail, Sac, and Duct tissues across the regulatory regions. As shown in Supplementary Fig. 15, the chromatin accessibility of 28 SpiDS genes was higher in the Sac than in the Tail and Duct (Supplementary Fig. 15a), but no such differences were obvious at the methylation levels (Supplementary Fig. 15c). A similar result also obtained for non-SpiDS genes (Supplementary Fig. 15b), possibly because more genes were expressed in the Sac (Fig. 2h). Notably, the main type of methylation was CG methylation, and the methylation level in gene regions was higher than that in gene flanking regions (Supplementary Fig. 15d).

Due to the identification of differential expression patterns in different parts of the Ma gland for *MaSp*-Group 1 and *MaSp*-Group 2 (Fig. 2h) as well as the observed chromatin accessibility patterns (Supplementary Fig. 16a,b), we screened these specific motifs for *MaSp*-Group 1 and *MaSp*-Group 2. The methods were as follows: 1) the chromatin accessibility peaks of the 2 kb upstream and 2 kb downstream regions of *MaSp1a–c* & *MaSp2e* and *MaSp2a–d* were filtered from the Tail, Sac, and Duct using the HOMER[55] tool, respectively; 2) for *MaSp*-Group 1, Tail- and Sac-enriched motifs, but not Duct-enriched motifs, were considered to be *MaSp*-Group 1-specific motifs; 3) for *MaSp*-Group 2, Sac-enriched motifs, but not Tail- and Duct-enriched motifs, were considered to be *MaSp*-Group 2-

specific motifs. We found that 9 and 13 motifs were significantly enriched in *MaSp1b* and *MaSp2b* (Supplementary Fig. 17a,b; Supplementary Data 15), respectively, and there were no identical motifs among them. These results revealed a common regulatory pattern within each of the *MaSp* groups but different regulatory patterns among the *MaSp* groups.

ceRNA network and expression analysis

We constructed the ceRNA network of 28 dragline silk genes by whole-transcriptome (WT) analysis to explore whether miRNAs and lncRNAs negatively regulate MaSps (see the text section "Comprehensive epigenetic features and ceRNA network of the tri-section Ma gland" for details). Specifically, the differential ceRNA network of *MaSp*-Group 1 and *MaSp*-Group 2 was constructed (Fig. 3g). Furthermore, we generated expression heatmaps of lncRNAs and miRNAs.

Difficulties in spatial sample preparation

Because the spatial morphology of the Ma gland is irregular, it is difficult to fix the Ma gland in a plane to ensure the integrity of the tissue sections. Specifically, the Tail, Sac, and Duct of the Ma gland are hollow (Supplementary Fig. 20a,b); the Duct is thin (diameter: ~100 mm) and soft; the Sac is enlarged, but the cell layer of the Sac is very thin; and the Tail is thin (diameter: ~300 mm) and curly. Importantly, the cell layer of the Sac is easy to peel off, and the tissue needs to be fixed in a 6.5 × 6.5 mm planar space. Based on the above factors, to capture more spatial information, we produced multiple slices to ensure that information was captured in all three parts, and the slice shown in Supplementary Fig. 20c was finally selected after many attempts. Although there was a small amount of loss in the Sac area, the contents of the Duct and Tail are captured within the 6.5 mm area, so we used this slice. For the ease of readers' understanding, we hand-painted the defective parts of this tissue section of interest without altering the authenticity of the data (Supplementary Fig. 20d).

Single-cell RNA-seq, ST clustering, and developmental trajectory analysis

To obtain high-quality single-cell (SC) data, a total of 9,349 cells were utilized for downstream analysis after quality control and doublets removal (Supplementary Fig. 19a,b). The "FindAllMarkers" function of the Seurat[56] package was used to find marker genes in each cluster. Due to there being no reference basis, the study is the first to report the SC types of the spider Ma gland. To distinguish cell types, we compared the scRNA-seq, ST, and bulk RNA-seq expression heatmaps of marker genes of each cluster according to the top 5 marker genes based on the scRNA-seq and ST analyses. The results showed a clear correspondence between heatmaps. Ten SC clusters were divided into Tail, Sac, and Duct parts (Supplementary Fig. 21a,b). For detailed descriptions, see the "Single-cell spatial architecture at the whole-Ma-gland scale" subsection of the main text.

The "DDRTree" method of the monocle[57] package was used to predict the developmental trajectories. To identify the starting point of cell development, we observed that the marker genes of cluster 1 were expressed in many other cell types, so we used cluster 1 as the cell starting point because the other two branches were the Duct and Tail branches (Fig. 4b, Supplementary Fig. 23), which were more specialized in function. Spatial spot clustering analysis also showed that the Tail, Sac, and Duct were divided into three distinct branches (Supplementary Fig. 24b).

Cell type annotation

To facilitate downstream analysis and understanding, we annotated all cell clusters according to the GO analysis of marker genes combined with the expression patterns of marker genes and segment-specific genes in each cluster. For cluster 1, as shown in Fig. 4b, most cells from cluster 1 assembled at the beginning of pseudotime and were defined as Ma gland origin cell (MaGO). For cluster 2, we defined it as the *MaSp*-Group 1 synthesis cell (MG1S) due to the high expression of *MaSp*-Group 1 genes (Fig. 4d) in this cluster and GO annotation as associated with ribosomes (the process of translation of RNA to protein occurs in the ribosome). For cluster 3, we first confirmed that this cluster belongs to the cell type of the Duct section based on the expression heatmap of marker genes in the scRNA-seq, ST, and bulk RNA-seq (Supplementary Fig. 21), and second that genes related to "Chitin binding" are highly expressed in this cluster (Fig. 4e), so we defined it as Chitin synthesis cell (CS). For cluster 4, it is difficult to speculate on the cell type with the available data, and it is named an unknown cell type. For cluster 5, the cluster was identified as Sac cell type based on marker genes expression patterns (Supplementary Fig. 21), the GO terms are enriched in "actin binding" and "cytoskeletal protein binding" and "actin cytoskeleton", we speculate that the swelling of the Sac portion may require a strong support force to prevent the Sac wall bursting by the luminal silk protein, so defined as ampullate lumen skeleton cell (ALS). For cluster 6, the GO terms were mainly enriched in "ATP metabolic process" and "generation of precursor metabolites and energy", and "calcium ion binding"-associated genes were highly expressed in this cluster (Fig. 4e), so they were named ion transport cell (IT). Cluster 7 was defined as lipid synthesis cell (LS) because of the high expression of lipid-related genes (Fig. 4e). For cluster 8, we found the highest expression of proton-transporting V-type ATPase-related genes in the cluster. Previous studies have also shown that V-type H+ ATPase-transport-related genes have a role in regulating pH values[58-60], and we named cluster 8 as the pH adjustment cell (PA). For clusters 9 and 10, we defined them as *MaSp*-Group 2 synthesis cell I (MG2S I) and *MaSp*-Group 2 synthesis cell II (MG2S II) because the *MaSp*-Group 2 genes are specifically expressed in cell clusters 9 and 10.

Dragline silk gene expression analysis in scRNA-seq and ST

Twenty-eight dragline silk proteins were identified in previous steps (Fig. 2h). Then, we focused on their expression levels in the scRNA-seq and ST results. The *MaSp*-Group 1 genes were found to be prominently highly expressed in clusters 1, 2, and 7 and weakly expressed in some other clusters (clusters 3, 5, 9, and 10), but the *MaSp*-Group 2 genes were found to be predominantly expressed within two unique cell clusters (clusters 9 and 10) (Fig. 4d). Accordingly, *MaSp*-Group 1 and *MaSp*-Group 2 showed similar results in the ST analysis; for example, *MaSp*-Group 1 genes were highly expressed in clusters "a, b, c, and d", and *MaSp*-Group 2 genes were highly expressed in cluster "d". These consistent results imply that different cell expression patterns exist between *MaS*p-Group 1 and *MaSp*-Group 2. Furthermore, we found that other silk genes were mainly expressed in clusters 1, 2, and 7 (Sac) (Supplementary Fig. 22b) and clusters "a, b, c, d, and e" (Sac) (Supplementary Fig. 22c), suggesting that other silk proteins are mainly produced in Tail and Sac.

KAT and Venom gene expression analysis based on ST

XA was shown to be the major pigment of golden dragline silk, and the kynurenine aminotransferase gene (KAT, Tc09G169510) encodes the primary enzyme catalyzing the transamination of 3-hydroxy-L-kynurenine (3-HK) to XA. Bulk RNA-seq analysis indicated that XA may be synthesized in the Tail, Sac, and Duct (Fig. 2i). According to the spatial expression plot of *KAT* in the Ma gland (Supplementary Fig. 27a), *KAT* was also expressed in all regions of the tissue, which further suggests that XA may be synthesized in all areas of the Ma gland. In addition, the Venom gene is expressed in the Tail and Sac of the Ma gland (Supplementary Fig. 27b) (clusters "a, b, c, and d"), which are also sites of *MaSp* group (Fig. 4d) but not of lipid genes (cluster "e"). Hence, we hypothesized that the function of this gene may be related to *MaSp*.

Transgenic vector construction and transgenic silkworm isolation

A nondiapausing silkworm strain, D9 L, was used in this study. Silkworm larvae were reared on fresh mulberry leaves under a 12 h light/12 h dark photoperiodic regime at 25 ± 1°C.

Transgenic overexpression of pierisin-1A in the MSG of silkworm. The piggyBac backbone vector with the Sericin1 promoter (piggyBac-*Ser1*P) was produced by our laboratory. The 7–807 bp pierisin-1A coding sequence was synthesized by the Gene Create company (China) and then cloned into the piggyBac-*Ser1*P backbone vector.

CRISPR-based marker gene knockout in silkworm. The Cas9 expression vector (piggyBac-hr3/A4-dCas9) and sgRNA backbone vector (piggyBac-U6-sgRNA) were produced by our laboratory. The sequence of sgRNA targeting *BMSK0007630* (homologous with *Tc04G175120*) was CTCGCCGCTCATTCAAGACGAGG.

Nondiapausing embryos of D9 L were collected immediately after oviposition. Transgenic vectors were microinjected into these embryos within 2 h using a micromanipulator (TransferMan NK2, Eppendorf) and a microinjector (Femto Jet 5247, Eppendorf) under a microscope (SZX16, Olympus). Economically important traits such as the cocoon weight, pupa weight, and cocoon layer ratio, of positive silkworm strains were investigated at the pupal stage. DNA from the positive silkworm strain was extracted and analyzed by deep sequencing[61].

Convergent evolution analysis

The transcriptomic data of the anterior part of the silk gland (ASG), the middle part of the silk gland (MSG), and the posterior part silk gland (PSG) of the silkworm and the proteomics and metabolomics data of cocoon silk were used for convergent evolution analysis compared with spider Ma gland and dragline silk. First, the orthologous gene pairs were identified between silkworm and spider by using the blastp[11] (E-value < $1e^{-5}$) program, resulting in 9,593 (*T. clavata*) – 7,355 (*B. mori*) one-to-multi orthologous genes being identified among them after deredundancy. The transcriptomic (ASG, MSG, and PSG) and genomic data of silkworm were downloaded from SilkDB3.0[62] (https://silkdb.bioinfotoolkits.net/) using the previous "Coexpression analysis" process for FPKM quantification. Differentially expressed genes (DEGs) were examined using the DESeq2[51] package. To screen for the unique genes of each part, we executed the following strategy: 1) orthologous gene expression convergence was analyzed (Supplementary Fig. 28a), wherein the overlapping URGs

between ASG vs. MSG and ASG vs. PSG were considered to be unique to the ASG (the unique genes of the MSG and PSG were defined similarly), and importantly, the overlapping URGs were considered orthologous genes between the silkworm and spider; 2) silk protein component convergence (Supplementary Fig. 28b) was analyzed, where the overlapping orthologous proteins between dragline silk and cocoon silk were identified; and 3) silk metabolite component convergence was analyzed, in which the overlapping metabolites between dragline silk and cocoon silk were identified.

A spider database resource: SpiderDB

Our exhaustive study of *T. clavata* has generated a wealth of omics data, including genomic, transcriptomic, epigenomics, SC data, and comparative genomics, etc. To better visualize these data at different biological levels, we developed SpiderDB (https://spider.bioinfotoolkits.net), an open and accessible database and powerful platform. SpiderDB is an online resource for presenting multiomics data of *T. clavata* in an interactive user interface. SpiderDB is mainly divided into the following modules: Gene-Info, eFP, Cell, Coexpression, SC, Gene Family, JBrowse, Chr, Synteny, and Ortholog (Supplementary Fig. 31). The Gene-Info module displays basic information about multiple aspects of the selected gene, such as gene ID, protein domain description, gene distribution, gene location, functional annotation, and sequences. The eFP (electronic fluorescent pictograph) viewer displays expression patterns by dynamically coloring the tissues represented by the pictograms according to gene expression levels, which is more intuitive and vivid. In Cell eFP, the database displays the predicted subcellular localization of proteins using ngLOC[63]. By analyzing the correlation between the expression levels of genes in different samples, a coexpression network between genes can be constructed so that the interaction relationship can be clarified. In the SC module, we used cellxgene to visualize gene expression and metadata annotation distributions across multiple datasets in the spider. The gene family module provides a user-friendly graphical view that displays the gene structure and Pfam domain pattern diagram linked to a bootstrapped similarity dendrogram to investigate the evolution of spiders, which contains a comprehensive gene comparison and evolution dataset with all the annotated genes in *A. bruennichi*, *D. silvatica*, *H. graminicola*, *O. gibbosus*, *T. antipodiana,* and *T. clavata*. The chromosome viewer provides an easy way to display the loci of selected genes and the gene families to which they belong on the 13 chromosomes. The synteny module shows the collinearity of chromosomes between *T. clavata* and other spiders. Ortholog module provides links to which orthologous clusters containing the selected gene. These modules take the user-interested gene as the keyword and integrate a variety of data visualization tools into the same interface, which makes it convenient for users to explore multilevel biological data and analyze genes comprehensively. SpiderDB is a web-based tool combining a MySQL database management system with a dynamic web interface that was written with Python, HTML, CSS, JavaScript, and jQuery. The entire project is openly available for anyone to use and was configured on an Ubuntu (V18.04) Linux machine with an Apache2 server. Considering the interactive user interface for the integration of large datasets, we think that the SpiderDB database will be a valuable resource for the spider research community.

Supplementary references

1. Agnarsson, I., Kuntner, M. & Blackledge, T.A. Bioprospecting finds the toughest biological material: extraordinary silk from a giant riverine orb spider. *PLoS One* **5**, e11234 (2010).
2. Gosline, J.M., Guerette, P.A., Ortlepp, C.S. & Savage, K.N. The mechanical design of spider silks: from fibroin sequence to mechanical function. *J. Exp. Biol.* **202**, 3295-303 (1999).
3. Heim, M., Keerl, D. & Scheibel, T. Spider silk: from soluble protein to extraordinary fiber. *Angew. Chem. Int. Ed. Engl.* **48**, 3584-96 (2009).
4. Marcais, G. & Kingsford, C. A fast, lock-free approach for efficient parallel counting of occurrences of k-mers. *Bioinformatics* **27**, 764-70 (2011).
5. Vurture, G.W. *et al.* GenomeScope: fast reference-free genome profiling from short reads. *Bioinformatics* **33**, 2202-2204 (2017).
6. Li, H. & Durbin, R. Fast and accurate long-read alignment with Burrows-Wheeler transform. *Bioinformatics* **26**, 589-95 (2010).
7. Li, H. *et al.* The Sequence Alignment/Map format and SAMtools. *Bioinformatics* **25**, 2078-9 (2009).
8. Zhu, B. *et al.* Chromosomal-level genome of a sheet-web spider provides insight into the composition and evolution of venom. *Mol. Ecol. Resour.* (2022).
9. Hendrickx, F. *et al.* A masculinizing supergene underlies an exaggerated male reproductive morph in a spider. *Nat. Ecol. Evol.* **6**, 195-206 (2022).
10. UniProt, C. UniProt: a hub for protein information. *Nucleic Acids Res.* **43**, D204-12 (2015).
11. Mount, D.W. Using the Basic Local Alignment Search Tool (BLAST). *CSH Protoc.* **2007**, pdb top17 (2007).
12. Cantalapiedra, C.P., Hernandez-Plaza, A., Letunic, I., Bork, P. & Huerta-Cepas, J. eggNOG-mapper v2: Functional Annotation, Orthology Assignments, and Domain Prediction at the Metagenomic Scale. *Mol. Biol. Evol.* **38**, 5825-5829 (2021).
13. Huerta-Cepas, J. *et al.* eggNOG 5.0: a hierarchical, functionally and phylogenetically annotated orthology resource based on 5090 organisms and 2502 viruses. *Nucleic Acids Res.* **47**, D309-D314 (2019).
14. Kanehisa, M. & Goto, S. KEGG: kyoto encyclopedia of genes and genomes. *Nucleic Acids Res.* **28**, 27-30 (2000).
15. Sneddon, T.P., Li, P. & Edmunds, S.C. GigaDB: announcing the GigaScience database. *Gigascience* **1**, 11 (2012).
16. Isard, M., Budiu, M., Yu, Y., Birrell, A. & Fetterly, D. Dryad: distributed data-parallel programs from sequential building blocks. *In Proceedings of the 2nd ACM SIGOPS/EuroSys European Conference on Computer Systems 2007* 59-72 (2007).
17. Members, C.-N. & Partners. Database Resources of the National Genomics Data Center, China National Center for Bioinformation in 2021. *Nucleic Acids Res.* **49**, D18-D28 (2021).
18. Pertea, G. & Pertea, M. GFF Utilities: GffRead and GffCompare. *F1000Res.* **9**(2020).
19. Emms, D.M. & Kelly, S. OrthoFinder: solving fundamental biases in whole genome comparisons dramatically improves orthogroup inference accuracy. *Genome Biol.* **16**, 157 (2015).
20. Edgar, R.C. MUSCLE: multiple sequence alignment with high accuracy and high throughput. *Nucleic Acids Res.* **32**, 1792-7 (2004).
21. Stamatakis, A. RAxML version 8: a tool for phylogenetic analysis and post-analysis of large phylogenies.

Bioinformatics **30**, 1312-3 (2014).

22. Kalyaanamoorthy, S., Minh, B.Q., Wong, T.K.F., von Haeseler, A. & Jermiin, L.S. ModelFinder: fast model selection for accurate phylogenetic estimates. *Nat. Methods* **14**, 587-589 (2017).
23. Yang, Z. PAML 4: phylogenetic analysis by maximum likelihood. *Mol. Biol. Evol.* **24**, 1586-91 (2007).
24. Kumar, S., Stecher, G., Suleski, M. & Hedges, S.B. TimeTree: A Resource for Timelines, Timetrees, and Divergence Times. *Mol. Biol. Evol.* **34**, 1812-1819 (2017).
25. Suyama, M., Torrents, D. & Bork, P. PAL2NAL: robust conversion of protein sequence alignments into the corresponding codon alignments. *Nucleic Acids Res.* **34**, W609-12 (2006).
26. Wang, D., Zhang, Y., Zhang, Z., Zhu, J. & Yu, J. KaKs_Calculator 2.0: a toolkit incorporating gamma-series methods and sliding window strategies. *Genom. Proteom. Bioinf.* **8**, 77-80 (2010).
27. Wang, Y. *et al.* MCScanX: a toolkit for detection and evolutionary analysis of gene synteny and collinearity. *Nucleic Acids Res.* **40**, e49 (2012).
28. Clarke, T.H., Garb, J.E., Hayashi, C.Y., Arensburger, P. & Ayoub, N.A. Spider Transcriptomes Identify Ancient Large-Scale Gene Duplication Event Potentially Important in Silk Gland Evolution. *Genome Biol. Evol.* **7**, 1856-70 (2015).
29. Langmead, B. & Salzberg, S.L. Fast gapped-read alignment with Bowtie 2. *Nat. Methods* **9**, 357-9 (2012).
30. Narasimhan, V. *et al.* BCFtools/RoH: a hidden Markov model approach for detecting autozygosity from next-generation sequencing data. *Bioinformatics* **32**, 1749-51 (2016).
31. Flynn, J.M. *et al.* RepeatModeler2 for automated genomic discovery of transposable element families. *Proc. Natl. Acad. Sci. USA* **117**, 9451-9457 (2020).
32. Xu, Z. & Wang, H. LTR_FINDER: an efficient tool for the prediction of full-length LTR retrotransposons. *Nucleic Acids Res.* **35**, W265-8 (2007).
33. Chen, N. Using RepeatMasker to identify repetitive elements in genomic sequences. *Curr. Protoc. Bioinformatics* **Chapter 4**, Unit 4 10 (2004).
34. Zhong, Y.F., Butts, T. & Holland, P.W. HomeoDB: a database of homeobox gene diversity. *Evol. Dev.* **10**, 516-8 (2008).
35. Thorvaldsdottir, H., Robinson, J.T. & Mesirov, J.P. Integrative Genomics Viewer (IGV): high-performance genomics data visualization and exploration. *Brief. Bioinform.* **14**, 178-92 (2013).
36. Ou, S. & Jiang, N. LTR_retriever: A Highly Accurate and Sensitive Program for Identification of Long Terminal Repeat Retrotransposons. *Plant Physiol.* **176**, 1410-1422 (2018).
37. Han, M.V., Thomas, G.W., Lugo-Martinez, J. & Hahn, M.W. Estimating gene gain and loss rates in the presence of error in genome assembly and annotation using CAFE 3. *Mol. Biol. Evol.* **30**, 1987-97 (2013).
38. Wu, T. *et al.* clusterProfiler 4.0: A universal enrichment tool for interpreting omics data. *Innovation (N Y)* **2**, 100141 (2021).
39. Supek, F., Bosnjak, M., Skunca, N. & Smuc, T. REVIGO summarizes and visualizes long lists of gene ontology terms. *PLoS One* **6**, e21800 (2011).
40. Chen, S., Zhou, Y., Chen, Y. & Gu, J. fastp: an ultra-fast all-in-one FASTQ preprocessor. *Bioinformatics* **34**, i884-i890 (2018).
41. Kim, D., Langmead, B. & Salzberg, S.L. HISAT: a fast spliced aligner with low memory requirements. *Nat. Methods* **12**, 357-60 (2015).
42. Langfelder, P. & Horvath, S. WGCNA: an R package for weighted correlation network analysis. *BMC*

Bioinformatics **9**, 559 (2008).
43. Minh, B.Q. *et al.* IQ-TREE 2: New Models and Efficient Methods for Phylogenetic Inference in the Genomic Era. *Mol. Biol. Evol.* **37**, 1530-1534 (2020).
44. Steentoft, C. *et al.* Precision mapping of the human O-GalNAc glycoproteome through SimpleCell technology. *EMBO J.* **32**, 1478-88 (2013).
45. Vollrath, F. Biology of spider silk. *Int. J. Biol. Macromol.* **24**, 81-8 (1999).
46. Chen, C. *et al.* TBtools: An Integrative Toolkit Developed for Interactive Analyses of Big Biological Data. *Mol. Plant* **13**, 1194-1202 (2020).
47. Wang, Z. *et al.* Chromosome-level genome assembly of the black widow spider Latrodectus elegans illuminates composition and evolution of venom and silk proteins. *Gigascience* **11**(2022).
48. Putthanarat, S. *et al.* The color of dragline silk produced in captivity by the spider Nephila clavipes. *Polymer* **45**, 1933-1937 (2004).
49. Hsiung, B.K., Justyn, N.M., Blackledge, T.A. & Shawkey, M.D. Spiders have rich pigmentary and structural colour palettes. *J. Exp. Biol.* **220**, 1975-1983 (2017).
50. Fujiwara, M. *et al.* Xanthurenic Acid Is the Main Pigment of Trichonephila clavata Gold Dragline Silk. *Biomolecules* **11**(2021).
51. Love, M.I., Huber, W. & Anders, S. Moderated estimation of fold change and dispersion for RNA-seq data with DESeq2. *Genome Biol.* **15**, 550 (2014).
52. Schultz, J., Copley, R.R., Doerks, T., Ponting, C.P. & Bork, P. SMART: a web-based tool for the study of genetically mobile domains. *Nucleic Acids Res.* **28**, 231-4 (2000).
53. Kumar, S., Stecher, G., Li, M., Knyaz, C. & Tamura, K. MEGA X: Molecular Evolutionary Genetics Analysis across Computing Platforms. *Mol. Biol. Evol.* **35**, 1547-1549 (2018).
54. Bailey, T.L. *et al.* MEME SUITE: tools for motif discovery and searching. *Nucleic Acids Res.* **37**, W202-8 (2009).
55. Heinz, S. *et al.* Simple combinations of lineage-determining transcription factors prime cis-regulatory elements required for macrophage and B cell identities. *Mol. Cell* **38**, 576-89 (2010).
56. Satija, R., Farrell, J.A., Gennert, D., Schier, A.F. & Regev, A. Spatial reconstruction of single-cell gene expression data. *Nat. Biotechnol.* **33**, 495-502 (2015).
57. Qiu, X. *et al.* Reversed graph embedding resolves complex single-cell trajectories. *Nat. Methods* **14**, 979-982 (2017).
58. Andersson, M. *et al.* Carbonic anhydrase generates CO2 and H+ that drive spider silk formation via opposite effects on the terminal domains. *PLoS Biol.* **12**, e1001921 (2014).
59. Wang, X. *et al.* Fiber Formation and Mechanical Properties of Bombyx mori Silk Are Regulated by Vacuolar-Type ATPase. *ACS Biomater. Sci. Eng.* **7**, 5532-5540 (2021).
60. Andersson, M., Holm, L., Ridderstrale, Y., Johansson, J. & Rising, A. Morphology and composition of the spider major ampullate gland and dragline silk. *Biomacromolecules* **14**, 2945-52 (2013).
61. Clement, K. *et al.* CRISPResso2 provides accurate and rapid genome editing sequence analysis. *Nat. Biotechnol.* **37**, 224-226 (2019).
62. Lu, F. *et al.* SilkDB 3.0: visualizing and exploring multiple levels of data for silkworm. *Nucleic Acids Res.* **48**, D749-D755 (2020).
63. King, B.R. & Guda, C. ngLOC: an n-gram-based Bayesian method for estimating the subcellular

proteomes of eukaryotes. *Genome Biol.* **8**, R68 (2007).

64. Ma, Y. *et al.* A single-cell transcriptomic atlas characterizes the silk-producing organ in the silkworm. *Nat. Commun.* **13**, 3316 (2022).
