# Supplementary material for: A molecular atlas reveals the tri-sectional spinning mechanism of spider dragline silk
Source: Nat Commun. 2023 Feb 15;14:837. doi: 10.1038/s41467-023-36545-6 (PMC9932165; doi:10.1038/s41467-023-36545-6)
Supplement: Supplementary file 2 — Reporting Summary [file 41467_2023_36545_MOESM2_ESM.pdf]

nature portfolio

Corresponding author(s): Yi Wang
Last updated by author(s): Dec 10, 2022

Reporting Summary

Nature Portfolio wishes to improve the reproducibility of the work that we publish. This form provides structure for consistency and transparency in reporting. For further information on Nature Portfolio policies, see our Editorial Policies and the Editorial Policy Checklist.

Statistics

For all statistical analyses, confirm that the following items are present in the figure legend, table legend, main text, or Methods section.

n/a	Confirmed	
☐	☒	The exact sample size (*n*) for each experimental group/condition, given as a discrete number and unit of measurement
☐	☒	A statement on whether measurements were taken from distinct samples or whether the same sample was measured repeatedly
☐	☒	The statistical test(s) used AND whether they are one- or two-sided<br>*Only common tests should be described solely by name; describe more complex techniques in the Methods section.*
☒	☐	A description of all covariates tested
☐	☒	A description of any assumptions or corrections, such as tests of normality and adjustment for multiple comparisons
☐	☒	A full description of the statistical parameters including central tendency (e.g. means) or other basic estimates (e.g. regression coefficient) AND variation (e.g. standard deviation) or associated estimates of uncertainty (e.g. confidence intervals)
☐	☒	For null hypothesis testing, the test statistic (e.g. *F*, *t*, *r*) with confidence intervals, effect sizes, degrees of freedom and *P* value noted<br>*Give P values as exact values whenever suitable.*
☒	☐	For Bayesian analysis, information on the choice of priors and Markov chain Monte Carlo settings
☒	☐	For hierarchical and complex designs, identification of the appropriate level for tests and full reporting of outcomes
☐	☒	Estimates of effect sizes (e.g. Cohen's *d*, Pearson's *r*), indicating how they were calculated

Our web collection on statistics for biologists contains articles on many of the points above.

Software and code

Policy information about availability of computer code

Data collection	Ubuntu 18.04<br>BLASTP 2.11.0+<br>Perl 5.26.2<br>Python 3.6.5<br>R 4.0.3<br>R studio 1.3.01093
Data analysis	Canu v2.2<br>Racon v1.5.0<br>Pilon v1.24<br>Hic-pro v2.10<br>3D-DNA v180419<br>Juicer v1.6.2<br>Juicebox v1.9<br>RepeatModeler v2<br>RepeatMasker v4.05<br>AUGUSTUS v3.2.3<br>MAKER2<br>MaxQuant v1.3.0.5

BSMAP 2.90
SAMtools v0.1.19
MACS2
Seurat v4.0.6
Monocle v2.22.0
More information and details were in the "Materials and methods" and "Supplementary Note".

For manuscripts utilizing custom algorithms or software that are central to the research but not yet described in published literature, software must be made available to editors and reviewers. We strongly encourage code deposition in a community repository (e.g. GitHub). See the Nature Portfolio guidelines for submitting code & software for further information.

Data

Policy information about availability of data

All manuscripts must include a data availability statement. This statement should provide the following information, where applicable:

- Accession codes, unique identifiers, or web links for publicly available datasets
- A description of any restrictions on data availability
- For clinical datasets or third party data, please ensure that the statement adheres to our policy

All high-throughput sequencing raw data in this project were deposited into the CNGB Nucleotide Sequence Archive (CNSA) of the China National GeneBank DataBase (CNGBdb, https://db.cngb.org) and are available through BioProject ID CNP0002864 (https://db.cngb.org/search/project/CNP0002864/). The mass spectrometry proteomics data have been deposited to the ProteomeXchange Consortium (http://proteomecentral.proteomexchange.org) via the iProX partner repository100 with the dataset identifier PXD038734. The single-cell and spatial metrices have been deposited in FigShare (https://figshare.com/articles/dataset/Single_cell_matrices_zip/20399475, https://figshare.com/articles/datasset/Spatial_Transcriptomics_zip/20399580). The genome assembly, gene annotation, and multiomics analysis results of Trichonephila clavata are also available on SpiderDB (https://spider.bioinfotoolkits.net).

Human research participants

Policy information about studies involving human research participants and Sex and Gender in Research.

Reporting on sex and gender	Not applicable.
Population characteristics	Not applicable.
Recruitment	Not applicable.
Ethics oversight	Not applicable.

Note that full information on the approval of the study protocol must also be provided in the manuscript.

Field-specific reporting

Please select the one below that is the best fit for your research. If you are not sure, read the appropriate sections before making your selection.

[x] Life sciences [ ] Behavioural & social sciences [ ] Ecological, evolutionary & environmental sciences

For a reference copy of the document with all sections, see nature.com/documents/nr-reporting-summary-flat.pdf

Life sciences study design

All studies must disclose on these points even when the disclosure is negative.

Sample size	The adult female Trichronephila clavata (body length without legs > 30 mm for each spider) was used in this study. The size of silk glands of adult female Trichronephila clavata ranges form 4 mm to 20 mm. Therefore, each gland/tissue sample was performed form 4-6 adult female spiders for per multiomics experiment, except for spatial transcriptomics (single silk gland required).
Data exclusions	No data were excluded from the analysis.
Replication	Each multiomics sample was performed form 4-6 adult female spiders. RNA sequencing (RNA-seq), liquid chromatography–mass spectrometry (LC–MS), the assay for transposase-accessible chromatin with sequencing (ATAC-seq), bisulfite-seq, and full RNA-seq were repeated at least in three replicated samples. Single-cell RNA seq of Ma gland used a mixed tissue sample from three adult female spider individuals. A total of 9,349 high-quality cells were generated. The scRNA-seq data can be verified with other multiomics data. Spatial transcriptomics used single Ma gland form a adult female spider individual. Because the spatial morphology of the Ma gland is irregular, it is difficult to fix the Ma gland in a plane to ensure the integrity of the tissue sections. Specifically, the Duct is thin (diameter: ~100 mm) and soft; the Sac is enlarged, but the cell layer of the Sac is very thin; and the Tail is thin (diameter: ~300 mm) and curly. Importantly, the cell layer of the Sac is easy to peel off, and the tissue needs to be fixed in a 6.5 × 6.5 mm planar space. Based on the above factors, to capture more spatial information, we produced multiple slices to ensure that information was captured in all three parts, and the slice shown in Supplementary Fig. 20c was finally selected after many attempts. Although there was a small amount of loss in the Sac area, the contents of the Duct and Tail are captured within the 6.5

	mm area, so we used this slice. For the ease of readers' understanding, we hand-painted the defective parts of this tissue section of interest without altering the authenticity of the data (Supplementary Fig. 20d).
Randomization	All samples were randomly allocated to experimental groups.
Blinding	Blinding dose not apply to this study. Blinding is not necessary because this study focuses on the multiomics of spider Ma gland.

Reporting for specific materials, systems and methods

We require information from authors about some types of materials, experimental systems and methods used in many studies. Here, indicate whether each material, system or method listed is relevant to your study. If you are not sure if a list item applies to your research, read the appropriate section before selecting a response.

Materials & experimental systems

n/a	Involved in the study	
☒	☐	Antibodies
☒	☐	Eukaryotic cell lines
☒	☐	Palaeontology and archaeology
☐	☒	Animals and other organisms
☒	☐	Clinical data
☒	☐	Dual use research of concern

Methods

n/a	Involved in the study	
☒	☐	ChIP-seq
☒	☐	Flow cytometry
☒	☐	MRI-based neuroimaging

Animals and other research organisms

Policy information about studies involving animals; ARRIVE guidelines recommended for reporting animal research, and Sex and Gender in Research

Laboratory animals	A nondiapausing silkworm (Bombyx mori) strain, D9 L, at the stage of the fifth instar and pupa was used in this study.
Wild animals	The adult golden orb-weaving spiders, Trichonephila clavata (basionym: Nephila clavata, Nephilidae, Trichonephila), were captured from the wild in Dali City, Yunnan Province, China. Spider samples were captured from web driectly by using bamboo stick. Tissues of all samples were dissected in PBS buffered saline and stored in 1.5mL microtubes at -80 °C until required.
Reporting on sex	All findings are applicable to female spiders, because a female orb-weaving spider can have up to seven morphologically differentiated types of silk glands.
Field-collected samples	The study did not involve samples collected form the field.
Ethics oversight	No ethical approval was obtained as it is not required for working on an invertebrate in China.

Note that full information on the approval of the study protocol must also be provided in the manuscript.
