# Supplementary material for: A molecular atlas reveals the tri-sectional spinning mechanism of spider dragline silk
Source: Nat Commun. 2023 Feb 15;14:837. doi: 10.1038/s41467-023-36545-6 (PMC9932165; doi:10.1038/s41467-023-36545-6)
Supplement: Supplementary file 4 — Description of Additional Supplementary Files [file 41467_2023_36545_MOESM4_ESM.pdf]

Description of Additional Supplementary Files

File Name: Supplementary Data 1

Description: Sample and sequencing Information

File Name: Supplementary Data 2

Description: Summary statistics for genome survey

File Name: Supplementary Data 3

Description: Summary statistics for *T. clavata* genome assembly

File Name: Supplementary Data 4

Description: Summary statistics for Transposon Elements

File Name: Supplementary Data 5

Description: Genome data resources for 12 species

File Name: Supplementary Data 6

Description: Homologous evidence for the identification of spider spidroin

File Name: Supplementary Data 7

Description: N- and C-terminal sequence for the identification of spider spidroin

File Name: Supplementary Data 8

Description: Motif classes statistics for 28 spidroins in *T. clavata*

File Name: Supplementary Data 9

Description: Statistics informations of the persent of strength, extensibility and stickiness related motifs

File Name: Supplementary Data 10

Description: Silk protein abundance and Ma tissue expression levels of 28 silk genes in the spider

File Name: Supplementary Data 11

Description: The FPKM expression ratio of 28 SpiDS genes

File Name: Supplementary Data 12

Description: Metabolite components of dragline silk

File Name: Supplementary Data 13

Description: Metabolite component categories in the dragline silk

File Name: Supplementary Data 14

Description: The GO enrichment results of the Duct, Sac, and Tail-specific expression genes in the transcriptome

File Name: Supplementary Data 15

Description: Specific motifs in the upstream or downstream 2 kb of *MaSp1b* and *MaSp2b*

File Name: Supplementary Data 16

Description: Different types of methylation ratios in different regions of the genome

File Name: Supplementary Data 17

Description: Marker genes in single-cell clusters

File Name: Supplementary Data 18

Description: Marker genes in ST clusters

File Name: Supplementary Data 19

Description: The top 5 GO annotations for each SC cluster

File Name: Supplementary Data 20

Description: Convergent evolution of genes between silkworm silk gland and spider Ma gland

File Name: Supplementary Data 21

Description: Silk protein abundance and silk gland tissue expression levels of 64 silk genes in silkworm

File Name: Supplementary Data 22

Description: Metabolite components of cocoon silk
